# A suberized exodermis is required for tomato drought tolerance

**DOI:** 10.1038/s41477-023-01567-x

**Published:** 2024-01-02

**Authors:** Alex Cantó-Pastor, Kaisa Kajala, Lidor Shaar-Moshe, Concepción Manzano, Prakash Timilsena, Damien De Bellis, Sharon Gray, Julia Holbein, He Yang, Sana Mohammad, Niba Nirmal, Kiran Suresh, Robertas Ursache, G. Alex Mason, Mona Gouran, Donnelly A. West, Alexander T. Borowsky, Kenneth A. Shackel, Neelima Sinha, Julia Bailey-Serres, Niko Geldner, Song Li, Rochus Benni Franke, Siobhan M. Brady

**Affiliations:** 1 Department of Plant Biology and Genome Center, University of California, Davis, Davis, CA USA; 2 Plant-Environment Signaling, Institute of Environmental Biology, Utrecht University, Utrecht, the Netherlands; 3 School of Plant and Environmental Sciences, Virginia Tech, Blacksburg, VA USA; 4 Department of Plant Molecular Biology, University of Lausanne, Lausanne, Switzerland; 5 Electron Microscopy Facility, University of Lausanne, Lausanne, Switzerland; 6 Institute of Cellular and Molecular Botany, Rheinische Friedrich-Wilhelms-University of Bonn, Bonn, Germany; 7 Department of Plant Biology, University of California, Davis, Davis, CA USA; 8 Center for Plant Cell Biology, Department of Botany and Plant Sciences, University of California, Riverside, Riverside, CA USA; 9 Department of Plant Sciences, University of California, Davis, Davis, CA USA; 10 Present Address: Department of Evolutionary and Environmental Biology, Faculty of Natural Sciences, Institute of Evolution, University of Haifa, Haifa, Israel

**Keywords:** Root apical meristem, Drought, Cell wall, Transcriptomics

## Abstract

Plant roots integrate environmental signals with development using exquisite spatiotemporal control. This is apparent in the deposition of suberin, an apoplastic diffusion barrier, which regulates flow of water, solutes and gases, and is environmentally plastic. Suberin is considered a hallmark of endodermal differentiation but is absent in the tomato endodermis. Instead, suberin is present in the exodermis, a cell type that is absent in the model organism *Arabidopsis thaliana*. Here we demonstrate that the suberin regulatory network has the same parts driving suberin production in the tomato exodermis and the *Arabidopsis* endodermis. Despite this co-option of network components, the network has undergone rewiring to drive distinct spatial expression and with distinct contributions of specific genes. Functional genetic analyses of the tomato MYB92 transcription factor and ASFT enzyme demonstrate the importance of exodermal suberin for a plant water-deficit response and that the exodermal barrier serves an equivalent function to that of the endodermis and can act in its place.

## Abstract (web summary)

Plant roots can respond to the environment by modifying cell type development. Here, the molecular pathways controlling root exodermal suberin are defined, as is its role in drought response. Modulating exodermal suberin levels can be a target for improved plant environmental resilience.

## Main

Plants have evolved complex cell type-specific regulatory processes to respond and adapt to dynamic environments. In certain cell types, such processes allow the formation of constitutive and inducible apoplastic diffusion barriers that regulate mineral, nutrient and water transport, pathogen entry, and have the capacity to alleviate water-deficit stress^1,2^. The *Arabidopsis thaliana* root endodermis contains both lignified and suberized diffusion barriers, of which the latter is extremely responsive to nutrient deficiency^3^. Many of the molecular players associated with suberin biosynthesis and the transcriptional regulation of this biosynthetic process have been elucidated using the *Arabidopsis* root endodermis as a model.

Suberin is a complex hydrophobic biopolymer, composed of phenylpropanoid-derived aromatic (primarily ferulic acid) and aliphatic (poly-acylglycerol) constituents, which is deposited between the primary cell wall and the plasma membrane as a lamellar structure^4,5^. While the order of the enzymatic reactions that produce suberin is not entirely understood^5^, many of the enzymes associated with suberin biosynthesis have been identified to function in the *Arabidopsis* root endodermis. These include the elongation of fatty acid acyl-CoA thioesters to very long chain fatty acid-CoA products by a fatty acid elongase complex for which the ketoacyl-CoA synthase (KCS) enzyme docosanoic acid synthase (DAISY) is the rate-limiting step^6^. Suberin primary alcohols are formed by the fatty acyl reductase (FAR) enzymes, which reduce C18:0-C22:0 fatty acids to primary fatty alcohols^7^. Suberin ω-hydroxyacids (ω-OH acids) and α,ω-dicarboxylic acids are produced by cytochrome P450 monooxygenases from the subfamilies CYP86A, CYP86B and CYP94B which are ω-hydroxylate fatty acids^4,8,9^. Glycerol esterification of fatty acid acyl-CoA derivatives is catalysed by the glycerol phosphate acyltransferases (GPAT) including GPAT5 (ref. ^10^). Ferulic acid is esterified to ω-hydroxyacids and primary alcohols by the feruloyl transferase ALIPHATIC SUBERIN FERULOYL TRANSFERASE (ASFT)/ω-HYDROXYACID/FATTY ALCOHOL HYDROXY-CINNAMOYL TRANSFERASE (FHT)^4,11,12^. Acyl-glycerol-esters are then subjected to transport into the apoplast and used as substrates for polymerization by GDSL-type esterase/lipase (GELP) enzymes^13^.

Many of the suberin biosynthetic enzymes acting in the root, periderm or seed were identified on the basis of their co-expression, leading to the hypothesis that a simple transcriptional module coordinates their transcription^4,8,14^. Although the overexpression of several transcription factors can drive suberin biosynthesis in either *Arabidopsis* leaves or roots^15,16^, the transcription of suberin biosynthetic genes is redundantly determined. It is only when a set of four *Arabidopsis* transcription factors—*MYB41, MYB53, MYB92* and *MYB93*—are mutated that suberin is largely absent from the *Arabidopsis* root endodermis^14^. Although not studied in roots, the *Arabidopsis* *MYB107* and *MYB9* transcription factors are required for suberin biosynthetic gene expression and suberin deposition in seeds^17,18^. These data demonstrate that multiple transcription factors coordinate the expression of suberin biosynthesis genes in *Arabidopsis*, dependent on the organ. Furthermore, components of these transcriptional regulatory modules are probably conserved across plant species, as orthologues of many of these transcription factors and their target genes are strongly co-expressed across multiple angiosperms^4,8,17^.

While the *Arabidopsis* root endodermis is well-characterized anatomically and molecularly, an additional root cell type deposits an apoplastic diffusion barrier during primary growth in other species^19^. This cell layer is found below the epidermis, is the outermost cortical cell layer of the root and has been referred to as either the hypodermis or the exodermis. The latter term was used given observations of a potential Casparian Strip (CS). Indeed, in 93% of angiosperms studied, the exodermal layer was reported to possess an apoplastic barrier composed of suberin or lignin^20^. Given the nature of these features, the exodermis is hypothesized to function similarly to the endodermis, although the need for two potential barrier layers is less clear^21,22^. The *Solanum lycopersicum* (tomato) root contains both an exodermis and an endodermis. At its first stage of differentiation, a lignified cap is deposited on the outmost (epidermal) face of exodermal cell walls as well as on its anticlinal walls. During its second stage of differentiation, suberin is deposited around the entire surface of the exodermal cells^23^. The drought or abscisic acid (ABA)-inducibility of tomato exodermal suberin is unknown as is the influence of root exodermal suberization on environmental stress responses. Given this similarity in timing and appearance of suberin between the tomato exodermis and *Arabidopsis* endodermis, two plausible hypotheses regarding their regulation are that they use the same regulatory networks or that they utilize distinct cell type-specific programmes. In the absence of a suberized endodermis, the plant may be more drought-susceptible, or the exodermal barrier may be sufficient to serve as the sole functional barrier.

To address these hypotheses, we profiled the transcriptional landscape of the tomato exodermis at cellular resolution and characterized suberin accumulation in response to the plant hormone ABA and in response to water deficit. We identified a co-expression module of potential suberin-related genes, including transcriptional regulators, and validated these candidates by generating multiple CRISPR–Cas9 mutated tomato hairy root lines using *Rhizobium rhizogenes*^24^ and tomato plants stably transformed with *Agrobacterium tumefaciens*, and screened them for suberin phenotypes using histochemical techniques. The validated genes included a MYB transcription factor (*SlMYB92, Solyc05g051550*) whose mutant has a reduction in exodermal suberin, and the *SlASFT* (*Solyc03g097500*) whose mutant has a disrupted exodermis suberin lamellar structure with a concomitant reduction in root suberin levels. To test the hypothesis that suberin is associated with tomato’s drought response, we exposed *slmyb92* and *slasft* mutant lines to water-deficit conditions. Both mutants displayed a disrupted response including perturbed stem water potential and leaf water status. This work describes a regulatory network with conserved parts and rewiring to yield distinct spatial localization, and contributions of specific factors to produce this environmentally responsive functional barrier.

## Results

### Development and composition of the suberized exodermis

**Fig. 1:**
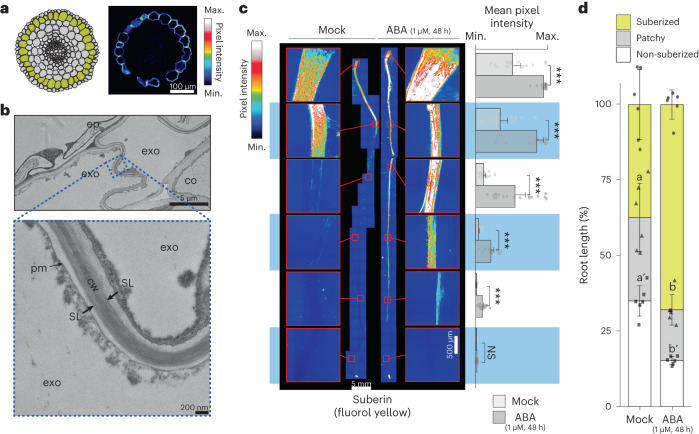
Suberin is deposited in the tomato exodermis and is regulated by ABA. **a**, Graphical representation (left) of *S. lycopersicum* (cv. M82) root anatomy (the exodermis is highlighted in yellow) and representative cross-section (right) of a 7-day-old root stained with FY. Scale bar, 100 µm. **b**, Transmission electron microscopy cross-sections of 7-day-old roots obtained at 1 mm from the root–hypocotyl junction. Top: the epidermal (ep), exodermal (exo) and inner cortex (co) layers. Bottom: a close-up of the featured region (zone defined with blue dotted lines), showing the presence of suberin lamellae (SL). cw, cell wall; pm, plasma membrane. **c**, Fluorol yellow (FY) staining for suberin in wild-type 7-day-old plants treated with mock or 1 µM ABA for 48 h. Whole-mount staining of primary root (left) and mean fluorol yellow signal along the root (right), *n* = 6; error bars, s.d. Asterisks indicate significance with one-way analysis of variance (ANOVA) followed by a Tukey-Kramer post hoc test (***P < 0.005). NS, not significant. **d**, Developmental stages of suberin deposition of wild-type plants treated with mock or 1 µM ABA for 48 h. Zones were classified as non-suberized (white), patchy suberized (grey) and continuously suberized (yellow); letters indicate statistically different groups; apostrophes indicate different statistical comparisons; *n* = 6; error bars, s.d.

**Extended Data Fig. 1:**
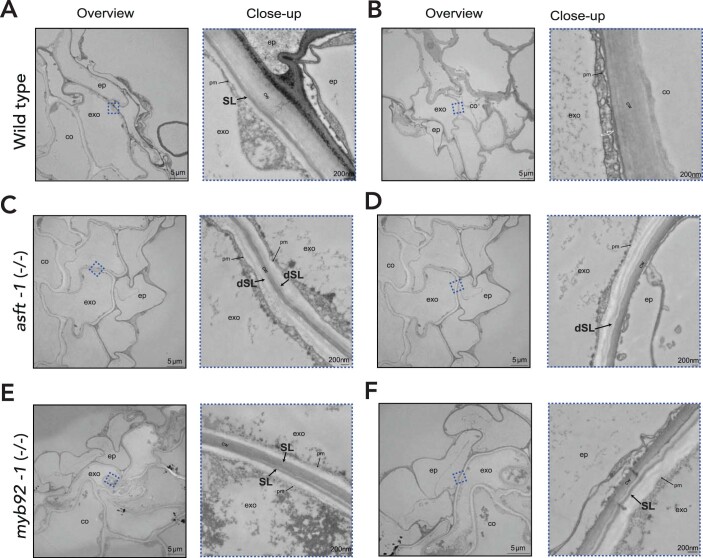
Suberin deposition and ultra-structure in tomato exodermal cells. Transmission electron microscopy cross-sections of 7-day-old wild type (a-b), *asft-1* (c-d) and *myb92-1* (e-f) plants obtained at 1 mm from the root-hypocotyl junction. Overview images show the epidermal, exodermal and inner cortex layers. Close-up images (corresponding to the zones defined with blue dashed lines in the adjacent Overview images) show the presence or absence of suberin lamellae. Black arrows indicate the presence of suberin lamellae, white arrow indicates areas where suberin lamellae could not be detected. scale bars = 5 µm for overview and 200 nm for close-up. Close-ups of c & f are repeated in Fig. 3. co = cortex, exo = exodermis, ep = epidermis, SL = suberin lamellae, dSL = defective suberin lamellae, cw = cell wall, pm = plasma membrane.

We previously quantified exodermis suberin deposition along the longitudinal axis of the tomato root (cv. M82, LA3475) using the histochemical stain Fluorol Yellow (FY). In *Arabidopsis* roots, suberin is absent from the endodermal cells in the root meristem and elongation zones, begins to be deposited in a patchy manner in the late differentiation zone after the CS has become established, and is then followed by complete suberization in the distal differentiation zone^25^. Quantification of exodermal suberin in 7-day-old tomato roots demonstrated the same three categories of deposition (none, patchy and complete) (Fig. 1a,c,d and Extended Data Fig. 1). Electron microscopy further demonstrated that within the completely suberized zone, suberin lamellae are deposited primarily on the epidermal and inter-exodermal faces of the exodermal cell (Fig. 1b and Extended Data Fig. 1). Suberin was consistently absent within the root endodermis throughout all developmental zones^23^ (Fig. 1a and Extended Data Fig. 1).

**Extended Data Fig. 2:**
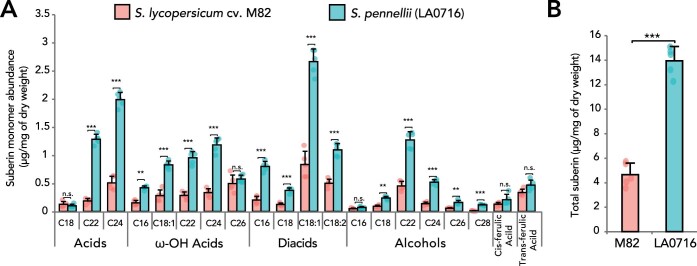
Roots of tomato wild relative *Solanum pennellii* (LA0716) contain significantly more suberin than *Solanum lycopersicum* (cv. M82). (**a**) Mean monomer abundance and (**b**) total suberin expressed as μg mg^−1^ of total dry weight. n = 5, error bars: SD. Asterisks indicate significance with one-way ANOVA followed by a Tukey-Kramer post hoc test: P: ‘***’ <0.001 ‘**’ <0.01, n.s.: ‘not significant’. Acid: fatty acids; Alcohols: primary alcohols; ω-OH: ω-hydroxy fatty acids; DCA: dicarboxylic fatty acid; Aromatics: ferulate and coumarate derivatives.

Monomer profiling of cell wall-associated and polymer-linked aliphatic suberin monomers in 1-month-old tomato roots revealed a predominance of α,ω-dicarboxylic acids, similar to potato^12^. Compared with *Arabidopsis* roots, which mostly feature ω-OH acids and a maximum chain length of 24 carbons (C24)^6,26^, additional C26 and C28 ω-OH acids and primary alcohols were observed in tomato (Extended Data Fig. 2a). This phenomenon of inter-specific variation in suberin composition has been previously observed^27^.

### Suberin biosynthetic enzymes and transcriptional regulators

**Extended Data Fig. 3:**
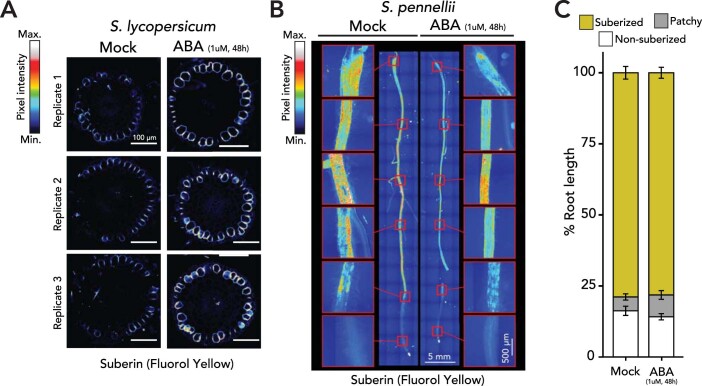
ABA treatment increases suberin levels but not localization in *Solanum lycopersicum*, while levels remain unchanged in *Solanum pennellii*. (**a**) Fluorol yellow staining of 7-day-old *S. lycopersicum* wild-type plants treated with mock or 1 µM ABA for 48 h. Cross-sections of roots at 1 cm from the hypocotyl junction. (**b**) Fluorol yellow staining for suberin in tomato wild relative *S. pennellii* (LA0716) 7-day-old plants treated with mock or 1 µM ABA for 48 h. Whole-mount staining of primary roots. (**c**) Developmental stages of suberin deposition of plants treated with mock or 1 µM ABA for 48 h. Zones were classified in non-suberized (white), patchy suberized (gray) and continuously suberized (yellow), n = 7, error bars: SD.

To map the tomato root suberin biosynthetic pathway and its transcriptional regulators, we leveraged previous observations of relative conservation of transcriptional co-regulation of the suberin pathway across angiosperms^4,8,17,28^. In the *Arabidopsis* root, suberin levels increase upon treatment with ABA^3,14^, a hormone which is a first responder upon water-deficit stress. Exodermal suberin deposition in tomato is similarly increased upon ABA treatment, both in terms of the region that is completely suberized as well as in the intensity of the signal (Fig. 1c,d), with the continued absence of endodermal suberin (Extended Data Fig. 3). *S. lycopersicum*’s wild relative, *Solanum pennellii* (LA0716), is drought tolerant^29–31^, and enhanced suberin deposition in *Arabidopsis* via mutation of *ENHANCED SUBERIN1* (*ESB1*) confers drought tolerance, although *esb1* also shows enhanced endodermal lignin and interrupted CS formation^1^. Hence, we tested and confirmed the hypotheses that *S. pennellii* has higher suberin deposition than M82 even in water-sufficient conditions and shows no changes in the magnitude or location of suberin deposition in response to ABA in seedlings (Extended Data Figs. 2 and 3). *S. pennellii* suberin levels are thus constitutive. Therefore, we utilized a gene expression dataset profiling transcription in M82 roots as well as across roots from 76 tomato introgression lines derived from *S. lycopersicum* cv. M82 and *S. pennellii* (LA0716) with M82 as the recurrent parent^32^. We additionally profiled the root transcriptomes of 1-month-old tomato plants under well-watered, waterlogged and water-deficit conditions. We hypothesized that genes directly involved in the biosynthesis and deposition of suberin will be highly correlated in both water-deficit and the introgression line population.

**Fig. 2:**
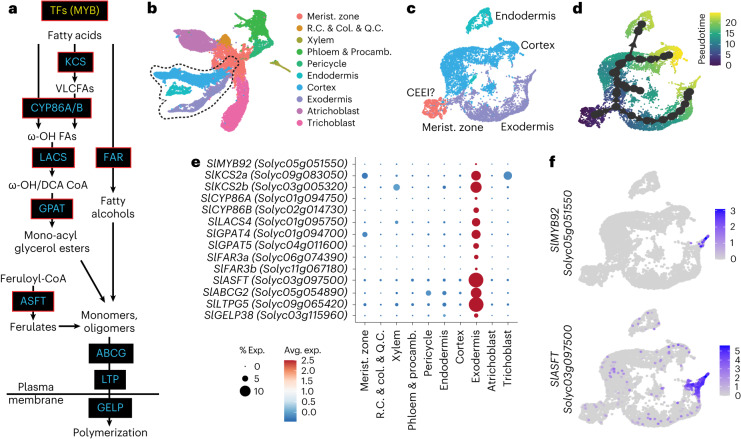
The tomato suberin biosynthetic enzymes and transcriptional regulator are expressed in the mature exodermis. **a**, Simplified diagram of the suberin biosynthesis pathway. Boxes indicate gene families involved in each step of the pathway (blue and yellow indicate biosynthetic enzymes and transcriptional regulators, respectively). Genes targeted in this study are outlined in red. TFs, transcription factors; VLCFAs, very-long-chain fatty acids. **b**, Annotated single-cell clusters from 3 cm of the tomato root tip displayed by an integrated UMAP. R.C., root cap. Q.C., quiescent centre; col, columella; procamb, procambium. **c**, UMAP of cortex/endodermis/exodermis-annotated cells that were extracted from the general projection and re-embedded. A small cluster of cells from the meristematic zone clusters were included to help anchor pseudotime estimations. CEEI, cortex–exodermis–endodermis initial. **d**, A pseudotime trajectory analysis for the cortex/endodermis/exodermis cell populations. **e**, Cell type or tissue-specific expression profiles for suberin biosynthetic pathway genes. Dot diameter represents the percentage of cells in which each gene is expressed (% Exp.) and colours indicate the average scaled expression of each gene in each developmental stage group with warmer colours indicating higher expression levels. **f**, Expression of *SlMYB92* and *SlASFT* in the single-cell transcriptome data. The colour scale represents log_2_-normalized corrected UMI counts.

By combining both introgression lines, waterlogging and water-deficit datasets in a weighted gene correlation network analysis (WGCNA)^33^, we identified modules of co-expressed genes (Supplementary Fig. 1). A module (‘royalblue’) containing 180 genes was significantly enriched in suberin-related genes (odds ratio = 16.1, *P* < 0.001). This was confirmed by intersection with a public dataset profiling gene expression in tomato *DCRi* lines (Supplementary Table 1). *DCRi* lines activate suberin-associated genes in the epidermal cells of fruit, which leads to suberization of the fruit surface^17^. The ‘royalblue’ module contains several orthologues of well-known suberin biosynthetic gene families such as glycerol-3-phosphate acyltransferases (GPATs), 3-ketoacyl-CoA synthases (KCSs) and feruloyl transferases (ASFT/FHTs) (Fig. 2a and Supplementary Table 1). In addition, putative tomato orthologues of known transcriptional regulators of suberin biosynthesis: *AtMYB41, AtMYB63* and *AtMYB92* (*SlMYB41*: *Solyc02g079280*; *SlMYB63*: *Solyc10g005550*; *SlMYB92*: *Solyc05g051550*), among others, were found in this module^14–16,34^.

### A single-cell tomato root atlas to map the exodermis

**Extended Data Fig. 4:**
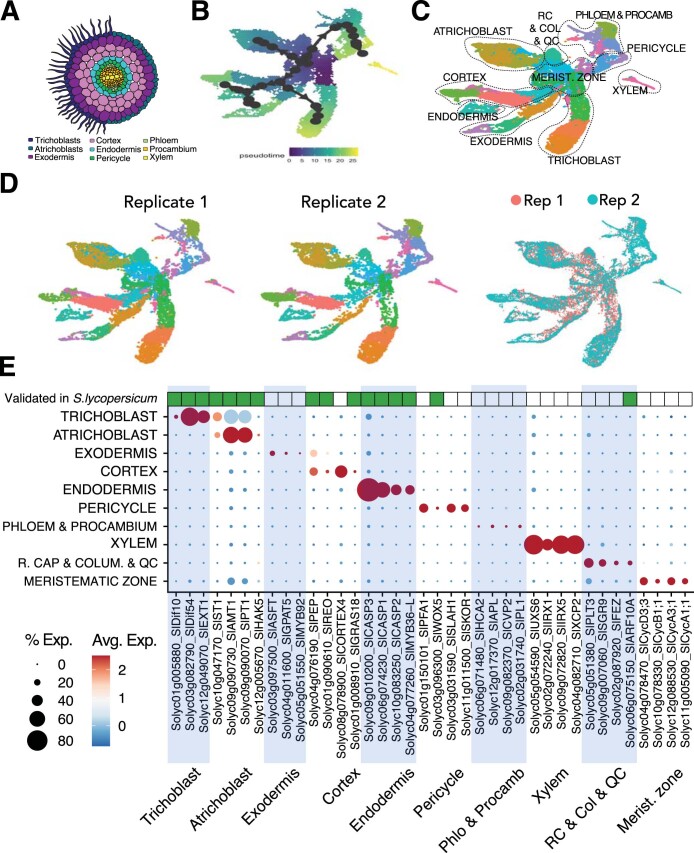
Single cell transcriptome atlas of the tomato root. (**a**) Graphical depiction of a tomato root section with cell types profiled in the single cell population. (**b**) A pseudo-time trajectory analysis ran on the population. (**c**) Annotation of single cell clusters displayed by an integrated uniform manifold approximation and projection (UMAP). Circles indicate subpopulations clustered together. (**d**) Reproducibility of biological replicates as observed by UMAP and cluster identification. (**e**) Expression profiles for 39 genes expressed across the major root tissue types. Dot diameter represents the percentage of cells in which each gene is expressed (% Exp.); and colors indicate the average scaled expression of each gene in each developmental stage group with warmer colors indicating higher expression levels. Top row indicates whether the gene’s expression has been validated in *S. lycopersicum* in previously published work. R.C.: Root cap. Q.C.: Quiescent center. Col: Columella. Procamb: Procambium. Phlo: Phloem.

Although translatome profiles exist for the exodermis^23^, these data do not provide resolution of the developmental gradient along which suberin is deposited. To refine the candidate suberin-associated gene set, we conducted single-cell transcriptome profiling of the tomato root. We used the 10X Genomics scRNA-seq platform to profile over 20,000 root cells. We collected tissue from 7-day-old primary roots of tomato (M82) seedlings up to 3 cm from the tip to include the region where suberin deposition is initially observed. Gene expression matrices were generated using cellranger and analysed in Seurat. Once the data were pre-processed and filtered for low-quality droplets, the remaining high-quality transcriptomes of 22,207 cells were analysed. After normalization, we used unsupervised clustering to identify distinct cell populations (Extended Data Fig. 4). These cell clusters were then assigned a cell type identity using the following approaches: We first quantified the overlap with existing cell type-enriched transcript sets from the tomato root^23^ and marker genes extracted from each of the clusters. An individual cluster was annotated as a specific cell type given the greatest overlap between the two sets and a significant adjusted *P* value (*P*_adj_ < 0.01). Then, to map gene expression dynamics across maturation, we examined cell-state progression by calculating pseudotime trajectories using a minimal spanning tree algorithm^35^. The tree was rooted in the root meristematic zone (identified in the previous step), and clusters were grouped into 10 cell types to reflect existing biological knowledge on differentiation of the tomato root (Fig. 2b and Extended Data Fig. 4). Lastly, genes with previously validated expression patterns in tomato^24,36–45^, transcriptional reporters^23^ and predicted cell type markers given their function in *Arabidopsis*^46^, were overlaid on the clusters to refine annotation (Extended Data Fig. 4c).

**Extended Data Fig. 5:**
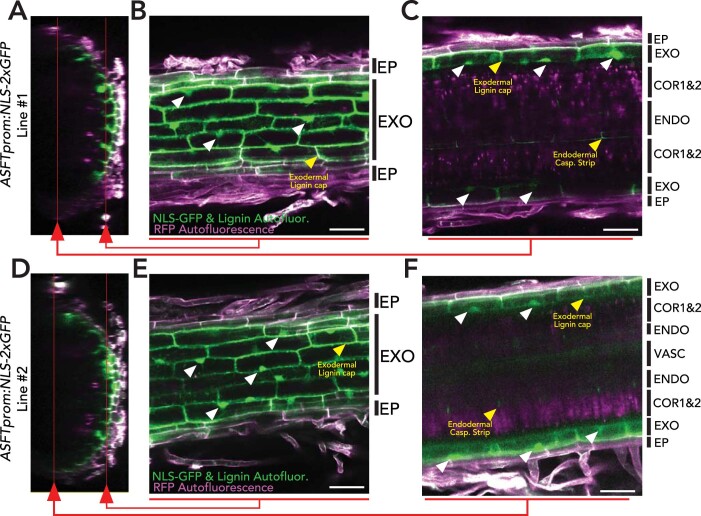
The *SlASFT* promoter drives exodermal-specific expression in roots. Representative images depicting expression of the *SlASFT* promoter driving nuclear localized GFP (*SlASFT*p:NLS-2xGFP) in two independently-transformed hairy root lines. NLS-GFP and lignin autofluorescence (Green) and epidermal RFP autofluorescence (Magenta). (A and D) Transversal z-stack projections of mature regions of transgenic hairy roots. Red lines indicate the planes shown in the subsequent longitudinal sections. (B and E) Longitudinal section of a top plane of the z-stacks, showing the epidermal and exodermal cell layers. White arrows indicate some of the GFP-tagged nuclei in the exodermis. Yellow arrows indicate the lignin autofluorescence of the exodermal polar cap. (C and F) Longitudinal section of a bottom plane of the z-stacks, showing the epidermis, exodermis, cortex layers 1 and 2, and endodermis. White arrows indicate the GFP-tagged nuclei in the exodermis. Yellow arrows indicate the lignin autofluorescence of the exodermal polar cap and the endodermal Casparian Strip. EP: Epidermis; EXO: Exodermis; COR1&2: Cortex layer 1 and 2; ENDO: Endodermis, VASC: Vasculature.

Given the successful annotation of these cell types, we focused on the mapped developmental trajectories deriving from a presumed cortex–endodermal–exodermal initial (CEEI) population (Fig. 2c,d). Within these three associated trajectories, we localized the cells in which the suberin biosynthetic enzymes and putative regulators were highly expressed (Supplementary Table 3). Of these, transcripts of *SlASFT* (*Solyc03g097500*), two *FAR* (*SlFAR3A*: *Solyc06g074390*; *SlFAR3B*: *Solyc11g067190*), two *CYP86* (*SlCYPB86A*: *Solyc01g094750*; *SlCYP86B1*: *Solyc02g014730*), two *KCS2* (*SlKCS2a*: *Solyc09g083050* and *SlKCS2b*: *Solyc03g005320*), two *GPAT* (*SlGPAT4*: *Solyc01g094700*; *SlGPAT5*: *Solyc04g011600*) and one *LACS* (*SlLACS4*: *Solyc01g095750*) showed restricted expression at the furthest edge of the exodermal developmental trajectory (Fig. 2e,f, and Supplementary Figs. 2 and 3). We generated a transcriptional reporter composed of the *SlASFT* promoter fused to nuclear-localized green fluorescent protein (GFP) and corroborated that its expression is restricted to the exodermis using hairy roots (Extended Data Fig. 5), in agreement with our single-cell analysis. In addition, of the three transcription factors previously noted (*SlMYB41*, *SlMYB63* and *SlMYB92*), only *SlMYB92* showed specific and restricted expression in cells at the tip of the exodermal trajectory (Fig. 2f and Supplementary Fig. 3). On the basis of the co-expression and cellular trajectory data, these genes served as likely candidates for an exodermal suberin transcriptional regulator and suberin biosynthetic enzymes.

### Knockout of candidate genes disrupt suberin deposition

**Fig. 3:**
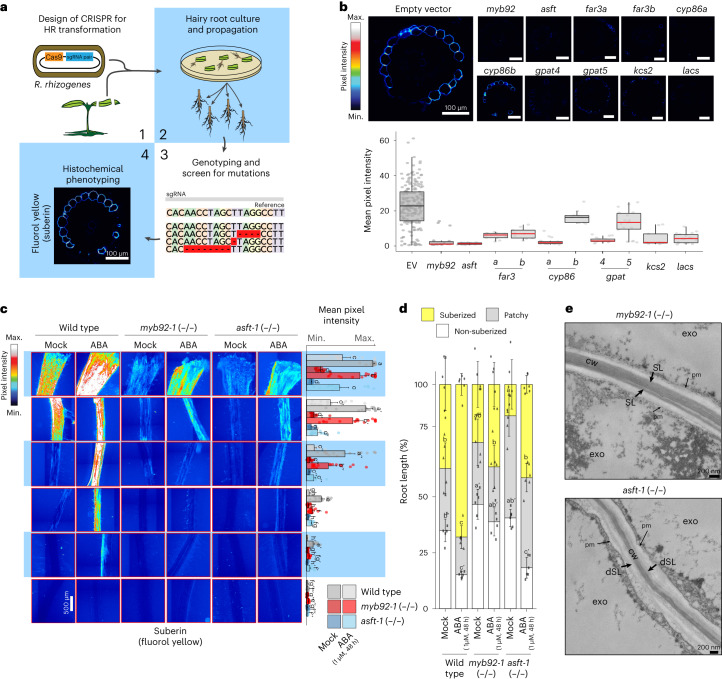
Loss-of-function mutant alleles of candidate genes disrupt exodermal suberization in tomato. **a**, Graphical summary of the hairy root (HR; *R. rhizogenes*) mutant screen. **b**, Summary of mutant phenotypes of candidate genes in hairy roots. Top: representative cross-sections of mature portions of the roots stained with fluorol yellow. Bottom: overall quantification of the fluorol yellow signal across multiple cross-sections (wild-type *n* = 66; rest *n* = 6). Red line indicates statistically significant differences in fluorol yellow pixel intensity in the mutant versus wild type as determined with a one-way ANOVA followed by a Tukey–Kramer post hoc test (*P*_adj_ < 0.05); EV, empty vector. Box plot centres depict the median while the bottom and top box limits depict the 25th and 75th percentile, respectively. Whiskers represent minima and maxima. Dots depict individual samples. **c**, Fluorol yellow staining for suberin in 7-day-old wild-type (repeated from Fig. 1 for reference), *slmyb92-1* and *slasft-1* plants treated with mock or 1 µM ABA for 48 h. Whole-mount staining of primary roots across different sections (left) and mean intensity of fluorol yellow signal along the root (right) (*n* = 6). Letters indicate significant differences (one-way ANOVA followed by a Tukey–Kramer post hoc test (*P*_adj_ < 0.05). Error bars, s.d. **d**, Developmental stages of suberin deposition in the 7-day-old wild-type and mutant plants treated with mock or 1 µM ABA for 48 h. Zones were classified as non-suberized (white), patchy suberized (grey) and continuously suberized (yellow) (*n* = 6). Letters indicate statistically different groups; apostrophes indicate different statistical comparisons **e**, Representative transmission electron microscopy cross-sections of *slasft-1* and *slmyb92-1* mutants obtained at 1 mm from the root–hypocotyl junction. The *slasft-1* mutant presents a deficit in suberin lamellar structure. cw, cell wall; dSL, defective suberin lamellae; exo, exodermis; pm, plasma membrane; SL, suberin lamellae.

**Extended Data Fig. 6:**
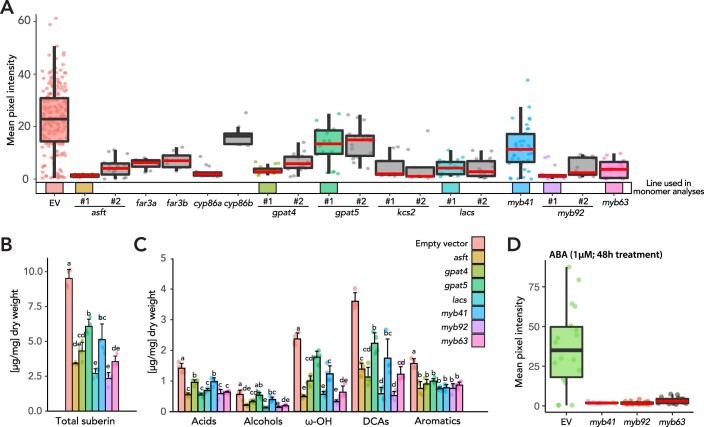
*R. rhizogenes*-derived loss-of-function mutant alleles of candidate genes have impaired suberin deposition. (**a**) Extended analysis of mutant phenotypes of candidate genes in hairy roots (HR). Mean fluorol yellow signal across multiple cross sections (wild type n = 66; rest n = 6). Red line indicates statistically significant difference (P < 0.05) in fluorol yellow pixel intensity in the mutant vs wild type as determined with a one-way ANOVA followed by a Tukey-Kramer post hoc test. In most cases, two independently generated HR lines were analyzed, as indicated in the plot. (**b**) Mean suberin abundance and (**c**) monomer composition of *R. rhizogenes*-generated mutants of suberin biosynthetic enzymes and transcriptional regulators. Acid: fatty acids; Alcohols: primary alcohols; ω-OH: ω-hydroxy fatty acids; DCA: dicarboxylic fatty acid; Aromatics: ferulate and coumarate derivatives. Error bars: SD. Letters indicate significant differences (one-way ANOVA followed by a Tukey-Kramer post hoc test, P < 0.05). (**d**) ABA treatment (1 µM for 48 h) does not restore suberin to wild type levels by fluorol yellow staining in *slmyb41*, *slmyb92* and *slmyb63* lines. Mean pixel intensities are not comparable between plots A and D as these were taken under different laser settings.

**Extended Data Fig. 7:**
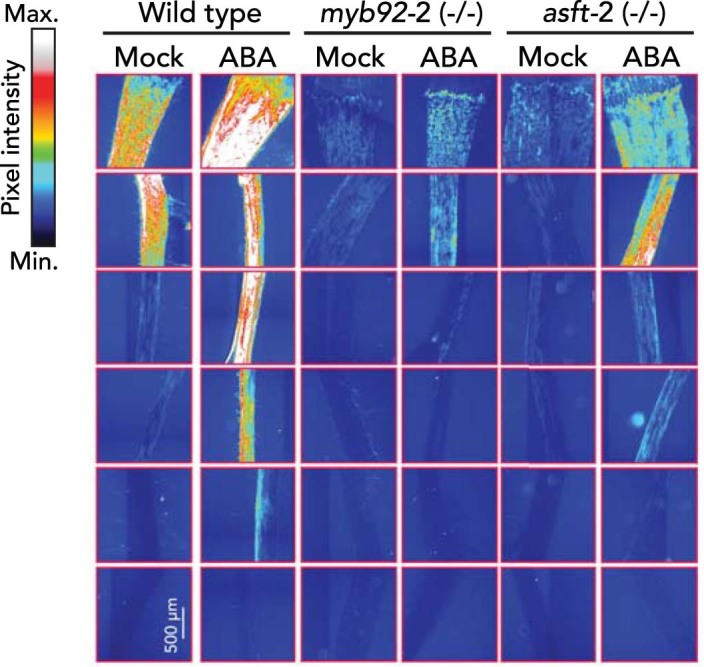
Impaired suberin deposition in the *myb92-2* and *asft-2* alleles and their impact on the response to ABA. Fluorol Yellow (FY) staining for suberin in wild type (repeated from Fig. 1 for reference), *myb92-2* and *asft-2* plants treated with mock or 1 µM ABA.

Functional validation of these enzymes was initially performed by CRISPR–Cas9 gene editing using two or three guide RNAs (Supplementary Table 4) and were introduced into tomato via *R. rhizogenes* (hairy root) transformation^24^ (Fig. 3a). Deletion-confirmed mutant alleles of these genes were phenotyped for suberin levels using fluorol yellow staining (Fig. 3b and Extended Data Fig. 6). On the basis of the histological phenotyping, all but the *slcyp86b* mutant showed a decrease in suberin (Fig. 3b). Further confirmation of decreased suberin levels were obtained by suberin monomer metabolic profiling in the *slgpat5*, *slgpat4*, *slasft*, *sllacs* and *slmyb92* mutants (Extended Data Fig. 6). These included collective reduction of ferulic acid and sinapic acid aromatic components; fatty acids (C20, C22, C24), ω-hydroxyacids (C18:2, C18:1, C20, C24, C26) and α-ω-diacids (C18:2, C18:1, C18, C20, C22). Given their expression in the terminus of the exodermal developmental trajectory (Fig. 2f), stable transgenic *slasft* and *slmyb92* deletion mutant alleles were generated by transformation with *A. tumefaciens* using the same guide RNAs. Recovered loss-of-function mutant alleles all had mutations leading to premature stop codons (Supplementary Table 4). Reduction of suberin levels as well as changes in its accumulation over the root developmental trajectory were observed in two independent mutant alleles of each gene (Fig. 3c,d and Extended Data Fig. 7). In the case of *slasft*, the significant delay in suberin deposition and changes in monomer composition in the exodermis differ from its orthologue in the *Arabidopsis* root endodermis, where the *atasft* mutant presents no defects in either deposition, timing or major monomer composition. The two mutants are similar in terms of their reduction in ferulate content^4,47^. We also observed disorganization of the lamellar structure in the *slasft-1* mutant, but not in the *slmyb92-1* mutant (Fig. 3e). While reduction of ferulic acids has been found in mutants of *ASFT* orthologues in potato^4,12^, this lamellar disorganization has not been reported before. Thus, while the enzymes responsible for suberin biosynthesis are largely the same for the tomato root exodermis and the *Arabidopsis* root endodermis, novelty is present in their contributions. That is, *SlASFT* is potentially directly responsible for both cell wall attachment and inter-lamella adhesion, and *MYB92* is a primary contributor to suberin deposition.

**Extended Data Fig. 8:**
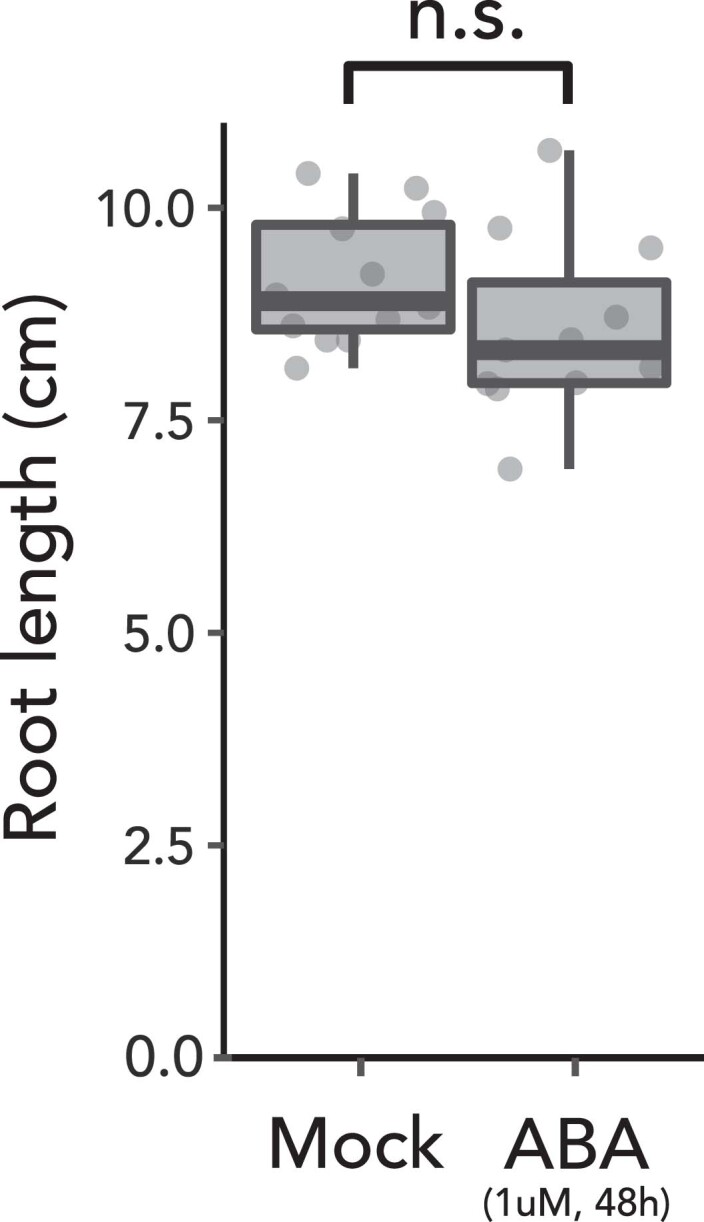
Root length is not significantly affected by the ABA treatment. Boxplot of total root length of 7-day-old wild-type plants treated with mock or 1 µM ABA for 48 h (n = 12). A one-way ANOVA analysis did not find any statistically significant differences.

**Extended Data Fig. 9:**
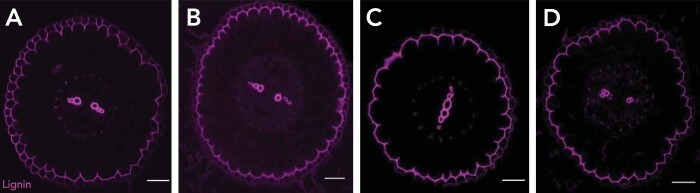
Lignin polar cap in the exodermis is not affected in the *myb41 myb63* and *myb92* hairy root mutants. **a**. Cross section of control hairy root stained with basic fuchsin. **b**. Cross section of *myb92* hairy root mutant stained with basic fuchsin. **c**. Cross section of *myb63* hairy root mutant stained with basic fuchsin. **d**. Cross section of *myb41* hairy root mutant stained with basic fuchsin. Scale bars=50 µm.

In *Arabidopsis*, ABA application increases suberin levels, and four MYB transcription factors are redundantly required for induction of ABA-mediated suberin accumulation in *Arabidopsis* (*AtMYB41*, *AtMYB53*, *AtMYB92* and *AtMYB93*)^3,14^. Given the ABA-inducibility of suberin in *Arabidopsis* and tomato (Fig. 1c,d)^1,3,48^, we hypothesized that *SlMYB92* or *SlASFT* are necessary for ABA-induced suberin biosynthesis. Therefore, we treated *slmyb92 and slasft* roots with 1 µM ABA for 48 h, a concentration that is sufficient to increase the completely suberized zone in tomato without perturbing root length (Fig. 1a and Extended Data Fig. 8). Although suberin levels were increased upon ABA treatment in these mutant backgrounds, both in the magnitude of the fluorol yellow signal and the proportion of the root that is completely suberized, the degree to which they were increased is reduced compared with wild type (Fig. 3c,d). This decrease in suberin levels in the *slmyb92-1* single mutant in both control and ABA-treated conditions is qualitatively greater than what was observed in the single mutant in *Arabidopsis*, which could be brought back to wild-type levels of endodermal suberin when exposed to ABA^14^. This difference suggests that SlMYB92 may play a more prominent role in suberin deposition than its *Arabidopsis* counterpart. The lack of this phenotype in the ABA-induced *atmyb92-1* mutant is explained by redundancy of the A*tMYB41*, *AtMYB53, AtMYB92* and *AtMYB93* transcription factors^14^. We explored whether such redundancy exists in tomato in the hairy root loss-of-function mutant alleles of *slmyb41* and *slmyb63*, and whether they were sufficient to decrease exodermal suberin in control and water-deficit conditions. ABA treatment was not able to induce suberin to wild-type levels in any of the mutants (Extended Data Fig. 6d). Since the exodermis is first lignified and then suberized^23^, we also explored whether *SlMYB92*, *SlMYB41* or *SlMYB63* were involved in lignification of the tomato root. However, the levels of lignin in the exodermis and endodermis remained unaffected in any of the hairy root mutants of these transcriptional regulators (Extended Data Fig. 9), suggesting that they do not have a role in the initial lignification of the exodermis or the endodermis.

### Impaired suberin deposition alters water limitation response

**Fig. 4:**
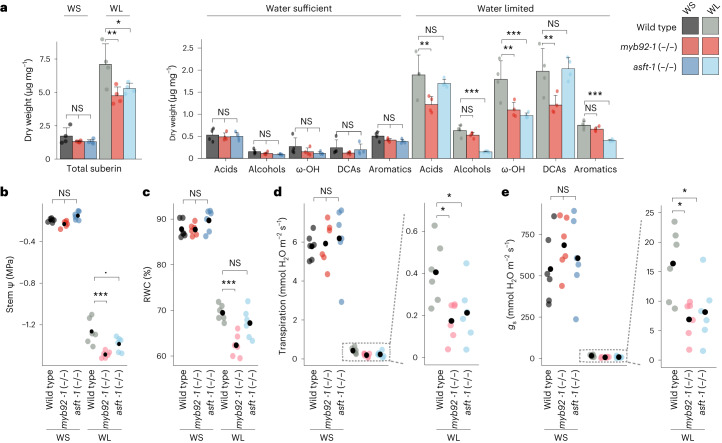
Impaired suberin deposition in *slmyb92-1* and *slasft-1* perturbs their whole-plant performances in response to water limitation. **a**, Suberin composition in roots of mature 1-month-old wild type, *slmyb92-1* and *slasft-1* plants. Plants were exposed to 10 days of water-sufficient (WS) and water-limitation (WL) regimes (*n* = 4, Methods). Acid, fatty acids; alcohols, primary alcohols; ω-OH, ω-hydroxy fatty acids; DCA, dicarboxylic fatty acid; aromatics, ferulate and coumarate isomers. Error bars denote s.d. **b**–**e**, Dot plots of recorded values for stem water potential (stem Ψ) (**b**), relative water content (**c**), transpiration (**d**) and stomatal conductance (*g*_s_) (**e**). Dotted line indicates zoom-in for better visual resolution of values. Black dots indicate mean values (*n* = 6). One-way ANOVAs for each treatment were performed followed by a Tukey–Kramer post-hoc test. ***P < 0.001 **P < 0.01 *P < 0.05 ‘.’ *P* < 0.1.

**Extended Data Fig. 10:**
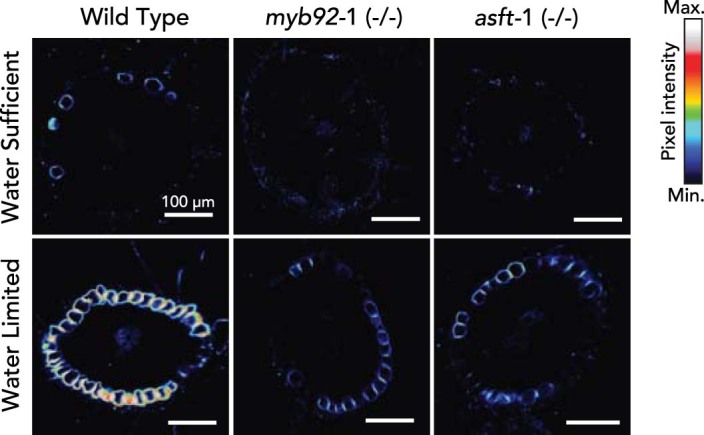
Suberin deposition in mature roots of wild type, *myb92-1*, and *asft-1*. Representative cross sections of mature portions of the roots stained with fluorol yellow (FY) of one-month-old plants exposed to 10 days of water sufficient (WS) and water limitation (WL) regimes (Methods).

Given the suggested link between suberin and drought tolerance, as well as the decreased suberin levels in both control and ABA conditions in our tomato mutants, we hypothesized that the *slmyb92* and *slasft* lines would be more sensitive to water limitation compared with wild-type plants. We subjected 4-week-old well-watered plants to 10 days of water-deficit conditions (Methods). Suberin deposition and monomer levels were studied in the root system of *slmyb92-1*, *slasft-1* and wild-type plants in both the water-sufficient and water-limited conditions. Under water-sufficient conditions, suberin deposition was only faintly observed in wild type, and exclusively in the exodermis, while being completely absent in both mutant lines (Extended Data Fig. 10). Consistent with this observation, very low levels of suberin monomers were detected, with no significant differences observed in the very long chain fatty acids, primary alcohols, ω-hydroxyacids, α-ω-dicarboxylic acids and aromatic components of suberin (Fig. 4a and Supplementary Fig. 4). Under water limitation, however, deposition of exodermal suberin was increased, with both mutant lines having lower levels than wild type (Extended Data Fig. 10). The transcriptional regulator mutant *slmyb92-1* showed a general reduction of most monomer groups compared with wild type. The *slasft-1* mutant, in comparison, was primarily depleted in ferulic acid and its esterification substrates, as well as in individual primary alcohols and ω-hydroxyacids (Fig. 4a and Supplementary Fig. 4). Furthermore, stem water potential, stomatal conductance and transpiration rate were significantly decreased in response to water-limited conditions in both *slmyb92-1* and *slasft-1* relative to wild type, and leaf relative water content was also decreased in *slmyb92-1* (Fig. 4b–e). When considering all physiological traits collectively using principal component analysis, *slasft-1* showed a milder water-deficit response compared with wild-type plants, while *slmyb92-1* was more extreme (Supplementary Fig. 5). These data demonstrate that decreased suberin levels in the tomato root exodermis directly perturb whole-plant performance under water-limited, but not under water-sufficient conditions. Furthermore, changes in specific suberin monomers and the lamellar structure that were observed between the two mutants in response to water-limited conditions may differently influence the extent of the physiological response.

## Discussion

In the well-characterized *Arabidopsis* root endodermis, suberin is deposited as a hydrophobic layer between the plasma membrane and the primary cell wall^5^. Developmentally, suberin biosynthesis and deposition occurs as a second step of endodermal differentiation, the first being the synthesis and deposition of the lignified Casparian strip^25^. Suberin serves as an apoplastic barrier and a transcellular barrier, thus contributing to the regulation of the movement of water and solutes to the vascular cylinder^49^. Our collective observations demonstrate that, relative to *Arabidopsis*, (1) the components of the pathway are conserved; (2) their spatial localization is distinct; (3) *ASFT* and *MYB92* are critical regulators of suberin biosynthesis given their phenotypes as single loss-of-function mutant alleles, as opposed to their redundancy in *Arabidopsis* and (4) exodermal suberin has equivalent function to endodermal suberin and can function in its absence (Fig. 1). Spatially, in the tomato root exodermis, suberin lamellae are deposited between the exodermal primary cell wall and the plasma membrane all around the cell, similar to the *Arabidopsis* root endodermis and other suberized apoplastic barriers such as the potato periderm^4^^,12^^,18^ (Fig. 1b). In a temporally similar fashion to the *Arabidopsis* endodermis, there is a non-suberized zone at the root tip, a patchy suberized zone in the middle of the root and a continuous suberized zone nearer to the root–hypocotyl junction (Fig. 1c,d).

We obtained clues to the underlying genes controlling exodermal suberin biosynthesis over developmental time by co-expression, single-cell transcriptome and genetic analyses. Conservation of the genes within the suberin biosynthetic pathway between *Arabidopsis* and tomato was evident from the functional genetic analysis of *SlCYP86B*, *SlGPAT*, *SlLACS* and *SlASFT* mutants. Despite the same genes controlling suberin biosynthesis, novelty in tomato is observed with respect to their tomato spatial (exodermal) expression and the critical contribution of *SlASFT* in primary cell wall attachment and inter-lamella adhesion of the suberin barrier. This phenotype has never been observed in *Arabidopsis* or potato *asft* mutant roots^4,12^. In addition, members of the GPAT4 subclade have been regarded as exclusively involved in cutin biosynthesis^50^, and here, *SlGPAT4* was shown to participate in the formation of exodermal suberin (Fig. 3). We focused on *SlMYB92* as a candidate due to its expression at the end of the exodermal trajectory. Although the precise timing of these trajectories is largely predictive in nature, we note that the expression of the biosynthetic enzymes does not completely overlap that of *SlMYB92* and suggests that SlMYB92 is not the sole transcriptional regulator of suberin gene expression. Our ability to obtain increasingly differentiated exodermal cells is probably limited by our ability to completely protoplast cells with secondary cell wall deposition. Therefore, the lack of *SlMYB41/53/93* expression in the exodermal trajectory does not mean that these genes are not expressed in the exodermis. In *Arabidopsis*, single loss-of-function mutants of *MYB41*, *MYB53* and *MYB93* show no changes in suberin levels, while that of *MYB92* shows a delay in suberization^14^. Extreme suberin phenotypes were only observed when mutations of all four genes were combined^14^. By contrast, this extreme phenotype (decrease of suberin levels in *slmyb92* mutant alleles as measured by fluorol yellow (Fig. 3b–d) and compositional profiling in hairy roots and stable lines (Extended Data Fig. 6 and Supplementary Fig. 4)) was observed in tomato when only MYB92 was mutated. The residual suberin levels found in the *slmyb92* mutants could be regulated by other MYB transcription factors. Indeed, mutants in tomato orthologues of *Arabidopsis* *MYB41* and *MYB63* showed exodermal suberin phenotypes (Extended Data Fig. 6), suggesting that these genes may be expressed in later exodermal developmental stages.

ABA-mediated regulation of tomato exodermal suberization is morphologically consistent with what is observed in the *Arabidopsis* root endodermis, with an increase in both the magnitude of suberin deposition and the proportion of the completely suberized zone^3,14^, despite the distinct spatial localization. At least in the case of the *slmyb92* and *slasft* mutant alleles and the ABA assays, this transcription factor and biosynthetic enzyme influence both developmental and ABA-mediated suberin deposition patterns (Fig. 3c,d). Further analyses of mutant alleles of the tomato *SlMYB41* and *SlMYB62* transcription factors will determine whether a coordinated developmental and stress-inducible regulation of suberin biosynthesis is the norm for exodermal suberin. The degree to which this regulation is dependent on ABA signalling, as it is in *Arabidopsis*^3^, also remains to be investigated. What remains to be identified, however, are the factors or regulatory elements that determine exodermal-specific regulation of these enzymes and transcriptional regulators, as well as how they are activated by ABA and why their activity is ABA-independent in *S. pennellii*.

External application of ABA can be considered a proxy for both drought and salt-stress response^51,52^. We tested the necessity of suberized exodermis for whole-plant performance under water-limited conditions in mature tomato plants (Fig. 4). The strongly reduced response of *slmyb92* and *slasft* plants to ABA was similarly observed upon drought stress. In both experiments, *slmyb92-1* and *slasft-1* failed to reach fluorol yellow signals and suberin levels equal to those of the control. Under control conditions (water-sufficient), we detected overall low suberin levels, which were near the detection limit of 0.003 µg mg^−1^ and reduced our ability to identify significant differences between the lines. This was consistent with the lack of distinct fluorol yellow signal in mature root sections under water-sufficient conditions (Extended Data Fig. 10). The effect observed in chemical suberin quantification may have also been attenuated by the sample comprising whole root systems with highly branched lateral roots and including root areas with immature suberin. *AtMYB92* is also known to regulate lateral root development in *Arabidopsis* together with its close orthologue *AtMYB93*^53^, and differences in suberin within different root types are a possibility. Regardless, suberin monomeric levels were clearly decreased in the *slmyb92-1* and *slasft-1* mutants in a distinct and overlapping fashion in response to water-limited conditions. Consistent with its function, *slasft-1* was primarily defective in accumulation of ferulate, primary alcohols and ω-hydroxyacids (Fig. 4a and Supplementary Fig. 4), while *slmyb92-1* had defects in fatty acids and the predominant unsaturated C18:1 ω-hydroxyacids and dicarboxylic acids (Fig. 4a and Supplementary Fig. 4). The more extreme perturbation of physiological responses in response to water limitation in *slmyb92-1* suggests that suberin composed of these fatty acid derivatives plays a role in controlling transcellular-mediated uptake of water (Fig. 4b,c). How the transcellular pathway operates in a root system where this apoplastic barrier is located four cell layers from the vascular cylinder remains an important and open question.

The role of exodermal suberin as an apoplastic barrier to water flow has been studied in maize and rice, where it was determined as a barrier to water flow, although maize and rice also present a suberized endodermis^54^. Thus, the role of exodermal suberin alone has never been studied with respect to its influence on plant responses to water limitation. The precise role of endodermal suberin, independent of the Casparian strip, has been studied in *Arabidopsis*, which lacks an exodermis^49^. In 21-day-old, hydroponically grown *Arabidopsis* plants, the *horst-1, horst-2, horst-1 ralph-1* and pCASP1:*CDEF1* mutants with a functional Casparian strip^49^ but with reduced suberin^8,9,25^ were monitored for the importance of suberin in water relations. These mutants, except for *horst-2*, have higher Lp_r_ (root hydraulic conductivity) and root aquaporin activity relative to wild type^49^. One can extrapolate that the decreases in stem water potential, transpiration and stomatal conductance relative to wild type in water-limited conditions (Fig. 4b,c) are a consequence of decreased suberin (*slmyb92*) or perturbations in suberin composition (*slasft*). Assuming that our suberin-defective mutants have higher root hydraulic conductivity^49^, our hypothesis to reconcile our observations with the higher Lpr would be that our mutants have compromised water-use efficiency under water limitation. This could lead to a delayed onset of the drought response such that the water loss is too great to recover by the time stomata are closed. The mechanisms by which this occurs need to be determined and could benefit from further exploration. The levels of lignin in the exodermis and endodermis were not altered in the mutants of the identified transcriptional regulators (Extended Data Fig. 9), and perturbations in endodermal lignin alone have no influence on root hydraulic conductivity in *Arabidopsis*, thus, lignin plays no role in our observations^49^.

To the best of our knowledge, the response of plants with decreased root exodermal suberin levels to water limitation has never been investigated. The importance of plant radial and cellular anatomy has also long been known as critical to our understanding of the role of plant roots in water uptake^55^ in the face of water deficit. Therefore, our findings provide direct evidence, via genetic perturbation, for the role of suberin in a specific cell type mediating tomato’s adaptive response to water deficit. Further, they impart a model by which exodermal suberin barriers contribute to whole-plant water relations in the absence of a suberized endodermis. While our findings are informative about the importance of suberin in the maintenance of transpiration and stomatal conductance under soil water deficit, our conclusions are limited to a particular stage of plant growth. Changes in response to water limitation in the field, particularly with genotypes with modified suberin that impart better maintenance of water potential, remains to be investigated.

Suberin in plants roots has recently been proposed to be an avenue to combat climate change including via sequestration of atmospheric CO_2_ as well as conferring drought tolerance^56^. This study provides evidence that root suberin is necessary for tomato’s response to water-deficit conditions. Increasing suberin levels within the root exodermis and/or the endodermis may indeed serve as such an avenue. The constitutive production of exodermal suberin in the drought-tolerant and wild relative of tomato, *S. pennellii* (Extended Data Fig. 3)^31,57,58^, certainly provides a clue that maintenance of suberin in non-stressed and stressed conditions may result in such a benefit. However, trade-offs of such an increase must also be considered. Increased suberin levels have been associated with pathogen tolerance^2,59,60^, but can also serve as a barrier to interactions with commensal microorganisms^61^ and constrain nutrient uptake, plant growth or seed dormancy^10,16,62^. Regardless, this complex process serves as an elegant example of how plant evolution has resulted in a gene regulatory network with the same parts but distinct spatial rewiring (exodermis instead of endodermis) and contributions of the different genes. Collectively, this rewiring results in the distinct but precise spatiotemporal biosynthesis and deposition of this specialized polymer to perform the equivalent function of endodermal suberin in a plant’s response to the environment.

## Methods

### Plant material and growth conditions

All tomato (*S. lycopersicum*) lines used in this study were derived from cultivar M82 (LA3475). The *S. pennellii* line used was LA0716. Seeds were surface sterilized in 70% (v/v) ethanol for 5 min followed by 50% (v/v) commercial bleach for 20 min and three washes with sterile deionized water. Seeds were plated on 12 cm× 12cm plates (without sucrose) or in Magenta boxes (with 30 g l^−1^ sucrose) containing 4.3 g l^−1^ Murashige and Skoog (MS) medium (Caisson, MSP09-50LT), 0.5 g l^−1^ MES (pH 5.8) and 10 g l^−1^ agar (Difco, 214530), and maintained in a 23 °C incubator with 16 h/8 h light/dark periods for 7–10 days until cotyledons were fully expanded and the true leaves just emerged and either harvested or transferred to soil.

### Tomato transformation

Hairy root transformants were generated on the basis of published work^24^. Stable transgenic lines were generated by *A. tumefaciens* transformation at the UC Davis Plant Transformation Facility.

### Transcriptome profiling of M82 roots under drought and waterlogging stress

Seedlings of *SlCO2p:*TRAP and *AtPEPp*:TRAP cv. M82 (ref. ^23^) were transplanted into 15 cm × 15 cm × 24 cm pots with Turface Athletic Profile Field & Fairway clay substrate (Turface Athletics) pre-wetted with a nutrient water solution (4% nitrogen, 18% phosphoric acid and 38% soluble potash). Plants were grown in a completely randomized design for 31 days in a growth chamber at 22 °C, 70% relative humidity, 16 h/8 h light/dark cycle and 150–200 mmol m^−2^ s^−1^ light intensity. For ‘well-watered’ conditions, we maintained substrate moisture at 40–50% soil water content. For water-deficit treatment, we withheld water from the plants for 10 days before harvest, and for waterlogged conditions, we submerged the pot until the root–shoot junction. We harvested the roots as close to relative noon as feasible (±2 h) by immersing the pot into cool water, massaging the root ball free, rinsing three times sequentially with water, dissecting the root tissues and flash-freezing with liquid nitrogen. We harvested the lateral roots (6–12 cm depth) and 1 cm root tips of adventitious roots. Sequencing libraries of adventitious roots were generated for each line in control and waterlogging conditions, and from lateral roots in control, waterlogging and water deficit conditions in four biological replicates per genotype/treatment, except for *SlCO2p*:TRAP lateral roots in control conditions (five biological replicates). Total RNA was isolated from these roots as previously described^63^, and non-strand specific random primer-primed RNA-seq library construction was performed as originally described^64^. RNA-seq libraries were pooled and sequenced with the Illumina HiSeq4000 (50SR).

### RNA-seq data processing and analysis for drought, water deficit and introgression line population

RNA-sequencing data processing and analyses for the drought, waterlogging and introgression line population were conducted as previously described^23^. Sequences were pooled, and then trimmed and filtered using TrimGalore! (v.0.6.6)^65^, with parameter -a GATCGGAAGAGCACA. Trimmed reads were pseudo-aligned to the ITAG3.2 transcriptome (complementary DNA) using Kallisto (v.0.43.1)^66^, with the parameters -b 100–single -l 200 -s 30, to obtain count estimates and transcripts per million (TPM) values. Samples were clustered with cuttreestatic^33^ and outliers removed (GSM2323699)^32^ with a minSize of 10.

### Generation of tomato CRISPR constructs

Guide RNAs (gRNAs) targeting exons were designed using the CRISPR-PLANT web tool (https://www.genome.arizona.edu/crispr/CRISPRsearch.html) (Supplementary Table 4). If this did not specify at least three guides with GC content between 40 to 60%, guides were designed with CRISPR-P V2 (http://crispr.hzau.edu.cn/cgi-bin/CRISPR2/CRISPR) using the U6 snoRNA promoter with <3 mismatches within the target gene coding sequence. Genomic sequences (ITAG3.2) were retrieved from Phytozome (https://phytozome-next.jgi.doe.gov/). Primer specificity was checked against *S. lycopersicum* RNA entries from NCBI’s RefSeq RNA. In cases where only two gRNAs were selected, olives containing gRNA sequence were ligated into pMR217/218 vectors and recombined via Gateway assembly into a pMR290 vector containing Cas9 and Kan resistance expression cassettes^67^. In cases where three gRNAs were selected, gRNAs were phosphorylated and ligated into pYPQ131-3 vectors, and then recombined into pYPQ143 via Golden Gate assembly. A pMR278 vector containing all three gRNA expression cassettes was then recombined into pMR286/289.

### Generation of the *SlASFT* transcriptional reporter construct driving a nuclear-localized GFP

A 2-kb region upstream of the ATG codon (oligos in Supplementary Table 5) was subcloned into D-TOPO and then recombined into pMK7FNFm14GW^68^.

### CRISPR–Cas9 mutant generation and analysis

Independently transformed lines were genotyped at the targeted genomic region (Supplementary Tables 4 and 5). In the case of hairy roots, at least 2 lines containing large deletions in both alleles in the gene of interest were kept for further analysis. In the case of stable transformants, first-generation (T_0_) transgenics were genotyped and self-pollinated. T_1_ plants with homozygous mutant alleles were selected. T_2_ and T_3_ were used in subsequent experiments.

### Water-deficit assay

Seedlings (7-day-old) were transferred to 0.5 l cones containing Turface pre-wetted with a nutrient water solution (containing 4% nitrogen, 18% phosphoric acid and 38% soluble potash). All pots were weight adjusted and a small set of pots were dried so that the percentage of water in the soil could be calculated. Plants were then grown in a completely randomized design for 3 weeks in a growth chamber at 22 °C, 70% relative humidity, 16 h/8 h light/dark cycle and ~150 μmol m^−2^ s^−1^ light intensity, and watered to soil saturation every other day. At the end of the first week, vermiculite was added to limit water evaporation from the soil. After 3 weeks, plants of each line were randomly assigned into two treatment groups (six plants each) and exposed to different treatments for 10 days. Control plants were watered to soil saturation with nutrient solution every day. Water-limited plants were exposed to water deficit by adjusting pot weights daily with nutrient solution (to the highest weight of the set) until a target soil water content of 40–50% was obtained. On the day of harvesting, between 09:00 to 12:00, stomatal conductance and transpiration were measured on the abaxial surface of the terminal leaflet of the third leaf or the youngest fully expanded leaf using a LICOR-6400XT portable photosynthesis system. Light intensity was kept at 1,000 µmol m^−2^ s^−1^, with a constant air flow rate of 400 µmol s^−1^ and a reference CO_2_ concentration of 400 µmol CO_2_ mol^−1^ air. The third (either left or right) primary leaflet was collected for measuring relative water content using a modified version of a previously established protocol^69^. Fresh leaves were cut with a scalpel leaving a 1-cm-long petiole and the total fresh weight (TFW) was measured. Leaves were then placed in individual zipper-locked plastic bags containing 1 ml of deionized water, making sure that only the leaf petiole is immersed in the solution. Bags were incubated at 4 °C. After 8 h, leaves were taken out of the bags, placed between two paper towels to absorb excess water and then weighed to determine the turgid weight (TW). Each sample was then placed into a paper bag and dried in a 60 °C dry oven for 3–4 days. Dried samples were weighed (DW), and relative water content was calculated as: RWC (%) = (TFW − DW) × 100/(TW − DW). A section of the fourth leaf, containing the terminal and primary leaflets, was used to measure stem water potential using a pump-up pressure chamber (PMS Instrument). The root systems were harvested by immersing the cone into water, massaging the root ball free, rinsing and removing excess water with paper towels. The middle section of the root system was sectioned using a scalpel. Around 300 mg of the dissected root tissue were added to Ankon filter bags (sealed with a staple). Bags were transferred into a glass beaker, an excess of chloroform:methanol (2:1 v/v) was added and extracted for 2 h. Fresh chloroform:methanol (2:1 v/v) was replaced and the extraction was repeated overnight under gentle agitation (twice). Fresh chloroform:methanol (1:2 v/v) was added and samples further extracted for 2 h. The extraction was repeated overnight twice with fresh chloroform:methanol (1:2 v/v). Finally, samples were extracted with methanol for 2 h. Methanol was removed and bags were dried in a vacuum desiccator for 72 h. Suberin monomer analysis was performed in these samples as described below.

### ABA assay

At 5 days after germination, seedlings from a plate were randomly transferred to fresh MS plates containing either 1 µM ABA or mock. After 48 h of treatments in a 23 °C incubator with 16 h/8 h light/dark, roots were harvested and used in subsequent analyses.

### Co-expression network analysis

Co-expression network modules were generated with the WGCNA (v.1.70). Libraries were quantile normalized and a soft threshold of 8 was used to create a scale-free network. A signed network was created choosing a soft thresholding power of 8, minModuleSize of 30, module detection sensitivity deepSplit of 2 and mergeCutHeight of 0.3. Genes with a consensus eigengene connectivity to their module eigengene of lower than 0.2 were removed from the module (minKMEtoStay). Modules were correlated with upregulated genes in DCRi lines described previously^17^ (Fisher’s exact test).

### Protoplast isolation and scRNA-seq

Seven days after sowing, 50–100 primary roots per sample of length ~3 cm from the root tip were cut and placed in a 35-mm-diameter dish containing a 70 μm cell strainer and 4.5 ml enzyme solution (1.25% w/v Cellulase R10, 1.25% Cellulase RS, 0.3% Macerozyme R10, 0.12% Pectolyase, 0.6 M mannitol, 20 mM MES (pH 5.7), 20 mM KCl, 10 mM CaCl_2_, 0.1% bovine serum albumin and 0.000194% (v/v) mercaptoethanol). Cellulase Onozuka R10, Cellulase Onozuka RS and Macerozyme R10 were obtained from Yakoult Pharmaceutical. Pectolyase was obtained from Sigma-Aldrich (P3026). After digestion at 25 °C for 2 h at 85 r.p.m. on an orbital shaker with occasional stirring, the cell solution was filtered twice through 40 μm cell strainers and centrifuged for 5 min at 500*g* in a swinging bucket centrifuge with the acceleration set to minimal. Subsequently, the pellet was resuspended with 1 ml washing solution (0.6 M mannitol, 20 mM MES (pH 5.7), 20 mM KCl, 10 mM CaCl_2_, 0.1% bovine serum albumin and 0.000194% (v/v) mercaptoethanol) and centrifuged for 3 min at 500*g*. The pellet was resuspended with 1 ml of washing solution and transferred to a 1.7 ml microcentrifuge tube. Samples were centrifuged for 3 min at 500 × *g* and resuspended to a final concentration of ~1,000 cells per μl. The protoplast suspension was then loaded onto microfluidic chips (10X Genomics) with v3 chemistry to capture 10,000 cells per sample. Cells were barcoded with a Chromium Controller (10X Genomics). Messenger RNA was reverse transcribed and Illumina libraries were constructed for sequencing with reagents from a 3’ Gene Expression v3 kit (10X Genomics) according to manufacturer instructions. Sequencing was performed with a NovaSeq 6000 (100-PE).

### Protoplasting-induced genes

Once protoplasts were purified, total RNA was extracted using the Direct-zol RNA miniprep kit (ZYMO). Bulk RNA-seq libraries were prepared using the QuantSeq 3’ mRNA-seq library prep kit FWD (Lexogen). Barcoded libraries were pooled, and PE 150-bp reads were sequenced on the NovaSeq 6000 instrument (Illumina) at the UC Davis DNA Technologies Core. Sequences were pooled, and then trimmed and filtered using TrimGalore! (v.0.6.6). R1 trimmed reads were pseudo-aligned to ITAG4.1 transcriptome (cDNA) using Kallisto (v.0.46.2), with the parameter -b 100, to obtain count estimates and TPM values. Differentially expressed genes with *P*_adj_ < 0.05 and logFC > 2 were selected as protoplast-induced genes (edgeR v.3.34.1).

### Single-cell transcriptome analysis

FASTQ files were mapped using cellranger (10X Genomics). Reads were aligned to the tomato genome (SL4.0) with the ITAG4.1 gene annotation file with organellar (mitochondria and plastid) sequences appended. Protoplasting-induced (Supplementary Table 2) genes, genes with counts in 3 cells or less, low-quality cells that contained <500 unique molecular identifiers (UMIs) and cells with >1% UMI counts belonging to organelle genes were filtered out. Data were then normalized using Seurat (v.4.0.5)^70^, followed by principal component analysis (PCA) and nonlinear dimensionality reduction using uniform manifold approximation and projection (UMAP). Fifty principal components were calculated and UMAP embedding was generated using the initial 35 principal components. Cluster-enriched genes were computed using the ‘FindAllMarkers’ function in Seurat using the only.pos = TRUE, min.pct = 0.1, logfc.threshold = 0.25 parameters.

### Single-cell cluster annotation

Clusters were annotated based on the overlap of cluster marker genes and a set of cell type-enriched marker genes from ref. ^23^. Given a set of tissue-specific markers for *T* number of tissue types, we call these sets *M*_*i*_ (*i* = 1…*A*), with *M*_*i*_ = {*m*_*i*1_, *m*_*i*2_… *m*_*i*n_}, *m*_*ij*_ representing genes in the marker list. These markers are mutually exclusive such that no genes appear in two different sets (*M*_*ij*_ ¹ *M*_km_ for any *i*, *k*). We identified the marker genes from Seurat-generated cluster markers *S*_*i*_ = {*s*_*i*1_, *s*_*i*2_, … *s*_*in*_}, (*i* = 1…*C*), where *C* equals the number of Seurat-generated clusters. We generated an overlapping table between *M*_*i*_ markers and *S*_*i*_ markers, which we represent in the table as *T*_*ij*_ (*i* = 1…*T*, *j* = 1…*C*). For each Seurat cluster, we hypothesized that the cells with the highest number of overlapping markers *T*_*ij*_ is the cell type of this cluster. A chi-squared test was used to determine the significance of overlap with Bonferroni correction:

1 $${\chi }^{2}=\varSigma \frac{{({O}_{i}-{E}_{i})}^{2}}{{E}_{i}^{2}}$$

with *i* = 1,2 and

*O*1 = number of highest overlapping markers argmax(*i*)*T*_*ij*_

*E*1 = expected number of overlapping markers sum (*T*_*ij*_)/*N*, *N* = number of tissue types

*O*2 = sum of markers that overlap with all other clusters sum (*T*_*ij*_) *i*¹imax

*E*2 = expected number of markers that overlap with all other clusters

This process was repeated for the second and third highest overlapping markers until the corrected *P* value was higher than 0.01. An individual cluster was assigned a tissue annotation type that had the most genes overlapping between the two marker sets, provided the *P*_adj_ value was significant for the overlap.

### Trajectory analysis

A trajectory analysis was run for the ground tissue cells^71^ after selecting and re-clustering the cell types annotated as exodermis and meristematic zone (clusters 0, 3, 8, 11, 12, 14, 15, 23, 25, 28). Gene expression matrices, dimensionality reduction and clustering were imported into the dynverse wrapper from Seurat and a starting cell was decided within the meristematic zone cluster. Trajectory inference was run using the minimum spanning tree (MST) algorithm. The MST method and UMAP coordinates from Seurat were used as input for mclust^72^. Predictive genes or genes that were differentially expressed along the trajectory, specific branches and milestones were identified and visualized with a heat map using dynfeature within the R package dynverse.

### Histochemical and imaging analysis

For sections, roots were divided in 1-cm segments, embedded in 4% agarose and sliced in 120-μm sections using a vibratome. Sections were then incubated in FY088 (0.01% w/v, dissolved in lactic acid) for 1 h at room temperature in darkness, rinsed three times with water and counterstained with aniline blue (0.5% w/v, dissolved in water) for 1 h in darkness. Confocal laser scanning microscopy was performed on a Zeiss Observer Z1 confocal microscope with the ×20 objective and GFP filter (488 nm excitation, 500–550 nm emission). For whole roots, suberin was observed in 7-day-old *S. lycopersicum* wild-type or mutant seedlings. Whole roots were incubated in methanol for 3 days, changing the methanol daily. Once cleared, roots were incubated in fluorol yellow 088 (0.05% w/v, dissolved in methanol) for 1 h at room temperature in the dark, rinsed three times with methanol and counterstained with aniline blue (0.5% w/v, dissolved in methanol) for 1 h at room temerature in the dark. Roots were mounted and observed with the EVOS cell imaging system (Thermo Fisher) using the GFP filter (488 nm excitation, 500–550 nm emission). Root sections were also stained with basic fuchsin (Fisher Scientific, 632-99-5). 1 cm segments from the root tip were embedded in 3% agarose and sectioned at 150–200 µM using a vibratome (Leica VT1000 S). The sections were stained in Clearsee with basic fuchsin for 30 min and then washed two times and imaged with a Zeiss LSM700 confocal microscope with the ×20 objective; basic fuchsin: 550–561 nm excitation and 570–650 nm detection. Hairy roots of *SlASFT* transcriptional fusions were imaged with the same confocal and objective, but with excitation at 488 nm and emission at 493–550 nm for GFP, and excitation at 555 nm and emission at 560–800 nm for red fluorescent protein (RFP) autofluorescence.

### Suberin monomer analysis

An average of 80 mg fresh weight root tissue per biological replicate (four biological replicates) was washed and immediately placed in a 2:1 solution of chloroform:methanol. Subsequently, root samples were extracted in a Soxhlet extractor for 8 h, first with CHCl_3_, afterwards with methanol to remove all soluble lipids. The delipidated tissues were dried in a desiccator over silica gel and weighed. Suberin monomers were released using boron trifluoride in methanol at 70 °C overnight. Dotriacontane was added to each sample (0.2 μg μl^−1^) as an internal standard, saturated NaHCO_3_ was used to stop the transesterification reaction, and monomers were extracted with CHCl_3_. The CHCl_3_ fraction was washed with water and residual water removed using Na_2_SO_4_. The CHCl_3_ fraction was then concentrated down to ~50 μl and derivatized with *N*,*N*-bis-trimethylsilyltrifluoroacetamide (BSTFA) and pyridine at 70 °C for 40 min. Compounds were separated using gas chromatography (GC) and detected using a flame ionization detector (6890 N Network GC System, Agilent Technologies) as previously described^26^. Compound identification was accomplished using an identical gas chromatography system paired with a mass spectroscopy selective detector (GC–MS; 5977A MSD, Agilent Technologies). Compounds were identified by their characteristic fragmentation spectra pattern with reference to an internal library of common suberin monomers and the NIST database.

### Transmission electron microscopy

Tomato roots were fixed in 2.5% glutaraldehyde solution (EMS) in phosphate buffer (PB 0.1 M; pH 7.4) for 1 h at room temperature and subsequently fixed in a fresh mixture of osmium tetroxide (1%, EMS) with 1.5% potassium ferrocyanide (Sigma) in PB buffer for 1 h. The samples were then washed twice in distilled water and dehydrated in acetone solution (Sigma) in a concentration gradient (30% for 40 min, 50% for 40 min, 70% for 40 min and 100% for 1 h three times). This was followed by infiltration in LR White resin (EMS) in a concentration gradient (33% LR White in acetone for 6 h, 66% LR White in acetone for 6 h and 100% LR White for 12 h two times) and finally polymerized for 48 h at 60 °C in an oven in atmospheric nitrogen. Ultrathin sections (50 nm) were cut transversely at 2, 5 and 8 mm from the root tip, the middle of the root and 1 mm below the hypocotyl–root junction using a Leica Ultracut UC7 (Leica Mikrosysteme), picked up on a copper slot grid 2 × 1 mm (EMS) and coated with a polystyrene film (Sigma). Micrographs and panoramic images were taken with an FEI transmission electron microscope (FEI CM100) at an acceleration voltage of 80 kV with a TVIPS TemCamF416 digital camera (TVIPS) using the software EM-MENU (v.4.0) (TVIPS). Panoramic images were aligned with the software IMOD (v.4.11)^73^.

### Phylogenetic tree construction

Phylogenetic trees were generated using the methods described in ref. ^23^.

### Statistics and reproducibility

All statistical analyses were done in the R environment (v.4.1.3), and derived plots done in ggplot2 (v.3.3.6). For multiple comparisons between genotypes, a one-way ANOVA was performed with a Tukey–Kramer post hoc test. Groups in which differences gave a *P* value lower than 0.05 were considered significantly different. All bar graphs represent mean, and error bars denote s.d. For all box plots, the centre depicts the median and the lower and upper box limits depict the 25th and 75th percentile, respectively. Whiskers represent minima and maxima. Closed dots depict individual samples. In all cases, individual biological samples are stated as n. Experiments and representative images were repeated independently at least three times, unless otherwise stated. Individual *P* values for all statistical analyses can be found in Supplementary Table 6.

### Reporting summary

Further information on research design is available in the Nature Portfolio Reporting Summary linked to this article.

### Supplementary information

Supplementary Information Supplementary Figs. 1–4.

Reporting Summary

Supplementary Table 1 Supplementary Tables 1–6.

## Data Availability

Single-cell and bulk RNA-seq data have been deposited at GEO (GSE212405) and have been made publicly available. CRISPR-generated mutant lines are available upon request. Timing is dependent upon obtaining phytosanitary certificates according to seed import regulations of the country of destination and associated costs.
