# Supplementary material for: A suberized exodermis is required for tomato drought tolerance
Source: Nat Plants. 2024 Jan 2;10(1):118–30. doi: 10.1038/s41477-023-01567-x (PMC10808073; doi:10.1038/s41477-023-01567-x)
Supplement: Supplementary file 1 — Supplementary Figs. 1–4. [file 41477_2023_1567_MOESM1_ESM.pdf]

nature plants

Article https://doi.org/10.1038/s41477-023-01567-x

A suberized exodermis is required for tomato drought tolerance

In the format provided by the authors and unedited

Supplemental Figures

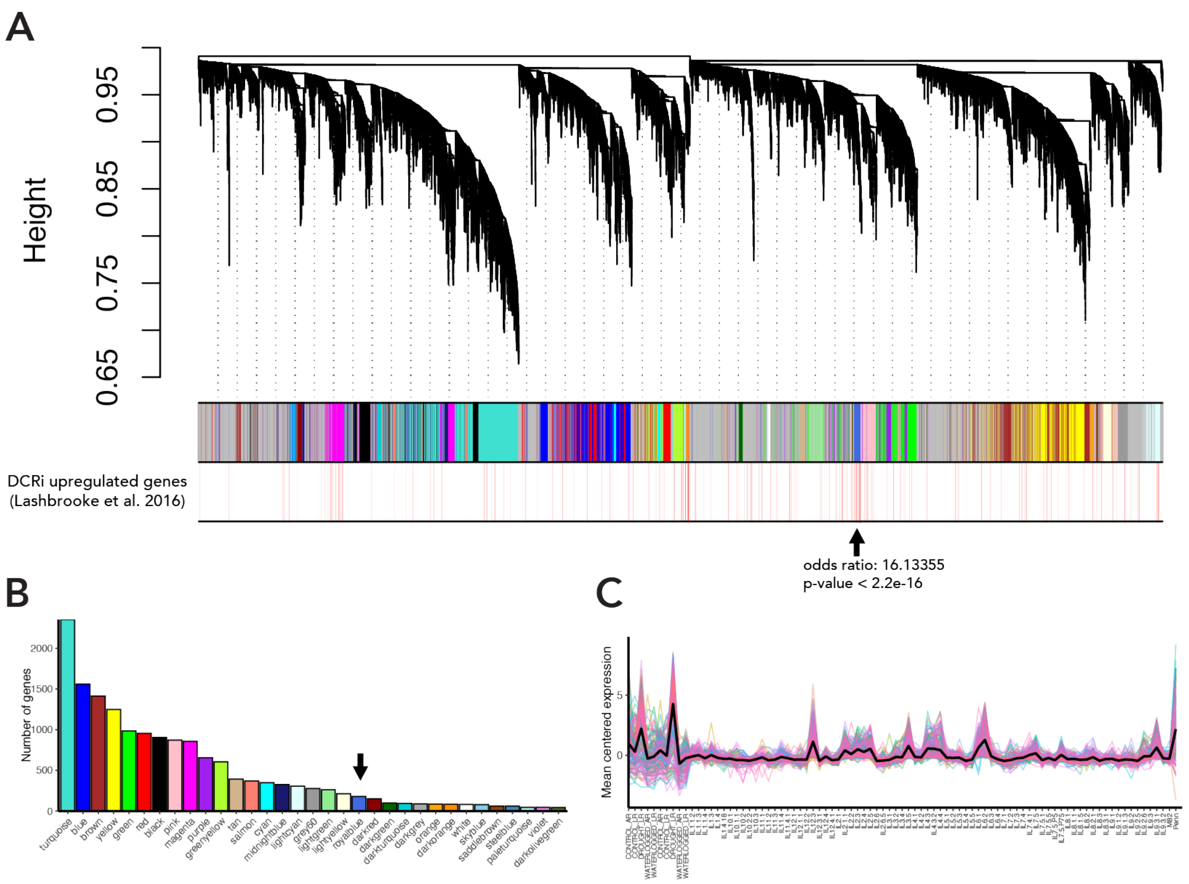

Supp. Fig. 1: WGCNA analysis of drought and IL datasets identifies a module enriched in suberin-related genes. (A) Gene dendrogram obtained by average linkage hierarchical clustering. The different colors underneath the dendrogram show module assignment as determined by the Dynamic Tree Cut. The bottom panel highlights (marked as thin red lines) the genes identified as upregulated in the suberin overexpression line DCRi in Lashbrooke et al. 2016. The "royal blue" (black arrow) module was significantly enriched for DCRi-upregulated genes. (B) Sizes of all gene modules identified in the analysis. The "royal blue" module (black arrow) contained 180 genes. (C) Mean centred gene expression of all the members of the "royal blue" module. Peaks in expression can be found in drought samples, in *Solanum pennellii* and in certain introgression lines.

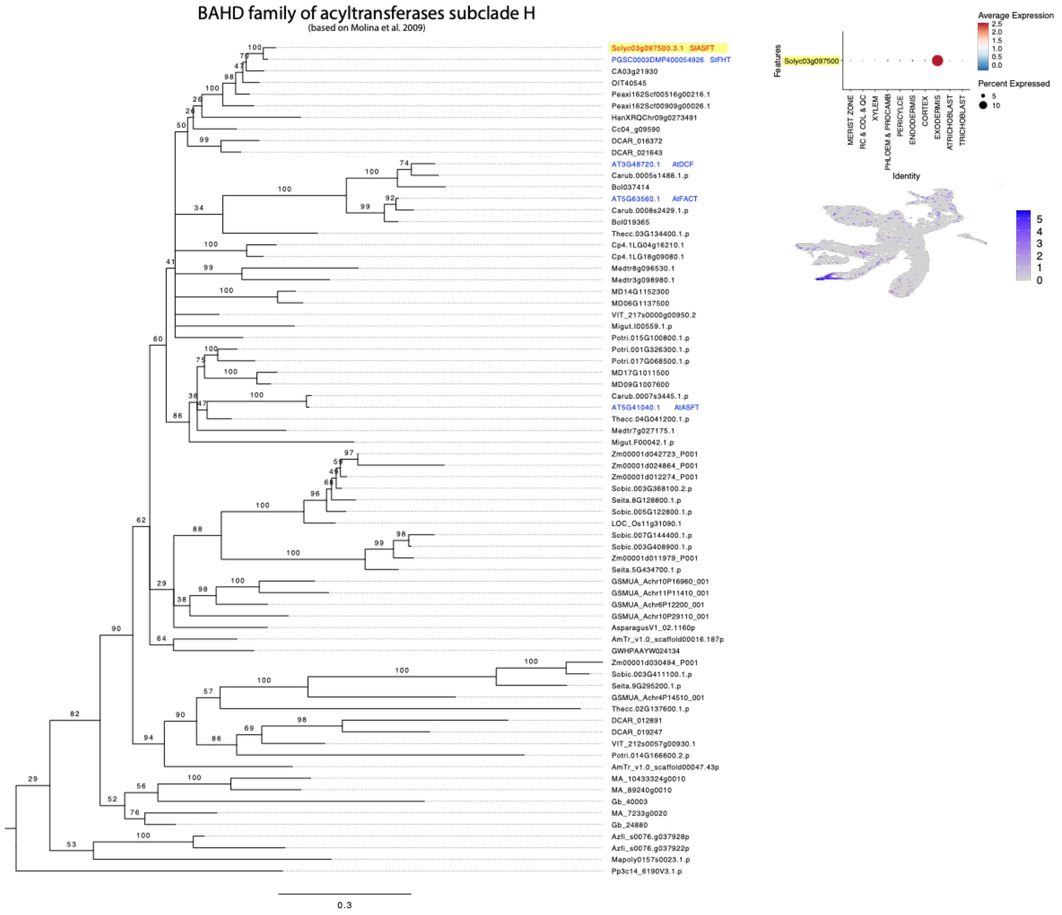

Supp. Fig. 2: Phylogenetic tree and single cell expression of potential suberin biosynthesis genes. (Left) Phylogenetic trees generated using protein sequences of several plant species. *S. lycopersicum* genes are highlighted in red. *A. thaliana* genes are highlighted in blue for reference. AmTr: *Amborella trichopoda*, AT: *Arabidopsis thaliana*, Asparagus: *Asparagus officinalis*, Azfi: *Azolla filiculoides*, Bol: *Brassica oleracea*, Carub: *Capsella rubella*, CA: *Capsicum annuum*, Cc: *Coffea canephora*, Cp: *Cucurbita pepo*, DCAR: *Daucus carota*, Gb: *Ginkgo biloba*, HanXRQ: *Helianthus annuus*, MD: *Malus domestica*, Mapoly: *Marchantia polymorpha*, Medtr: *Medicago truncatula*, Migut: *Mimulus guttatus*, GSMUA: *Musa acuminata*, OIT: *Nicotiana attenuata*, GWHPAAYW: *Nymphaea colorata*, LOC_Os: *Oryza sativa japonica*, Peaxi: *Petunia axillaris*, Pp: *Physcomitrella patens*, MA: *Picea abies*, Potri: *Populus trichocarpa*, Semoe: *Selaginella moellendorffii*, Seita: *Setaria italica*, Solyc: *Solanum lycopersicum*, PGSC: *Solanum tuberosum*, Sobic: *Sorghum bicolor*, Thecc: *Theobroma cacao*, VIT: *Vitis vinifera*, Zm: *Zea mays*. (Right Top) Expression profiles for genes of the suberin biosynthetic pathway. Dot sizes represent the percentage of cells in which each gene is expressed (% Exp.); and colors indicate the average scaled expression of each gene in each developmental stage group with warmer colors indicating higher expression levels. R.C.: Root cap. Q.C.: Quiescent center. Col: Columella. Procamb: Procambium. (Right bottom) Expression of *S. lycopersicum* genes in the single cell population. The color scale represents log2-normalized corrected UMI counts.

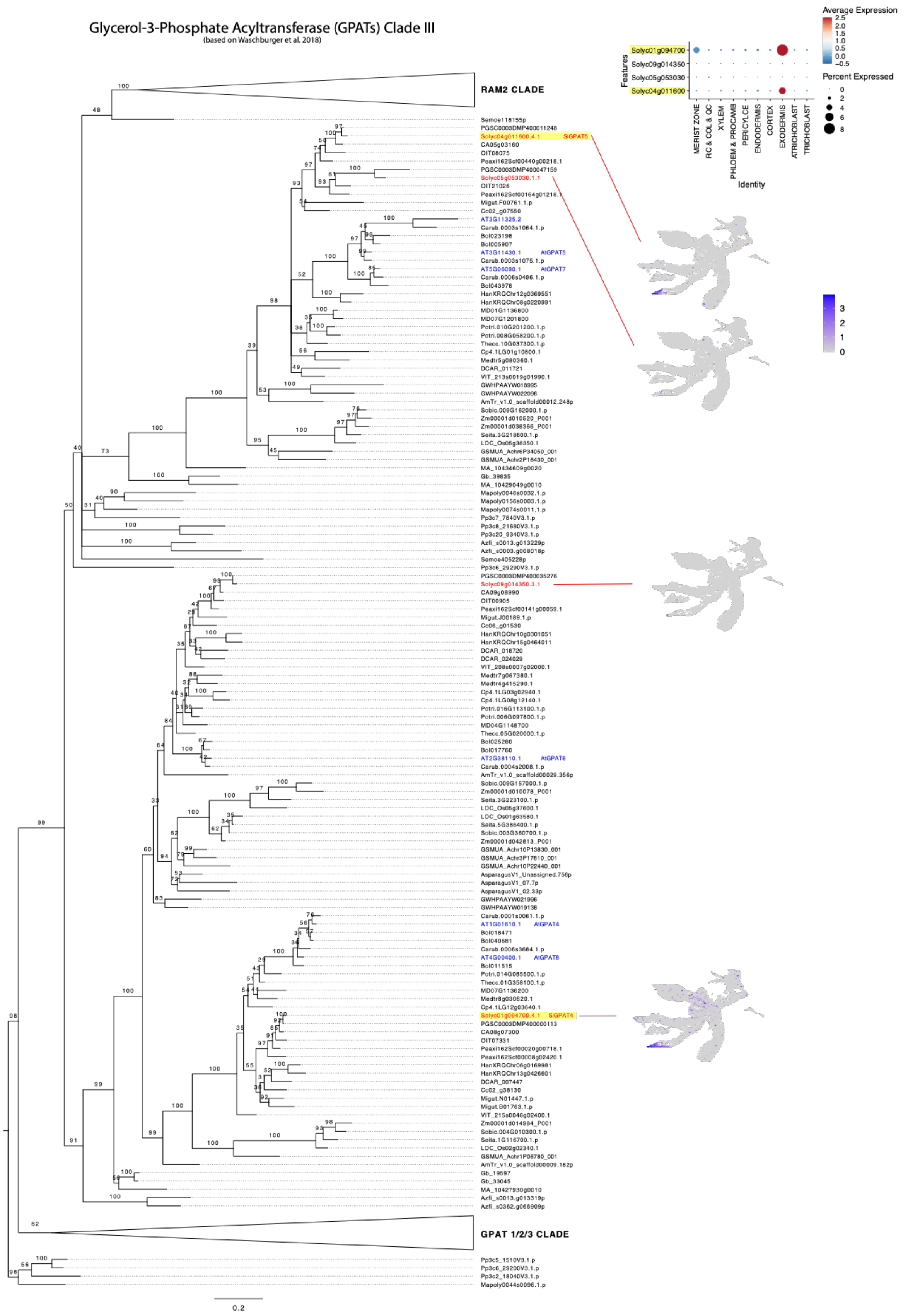

Supp. Fig. 2 (Cont.)

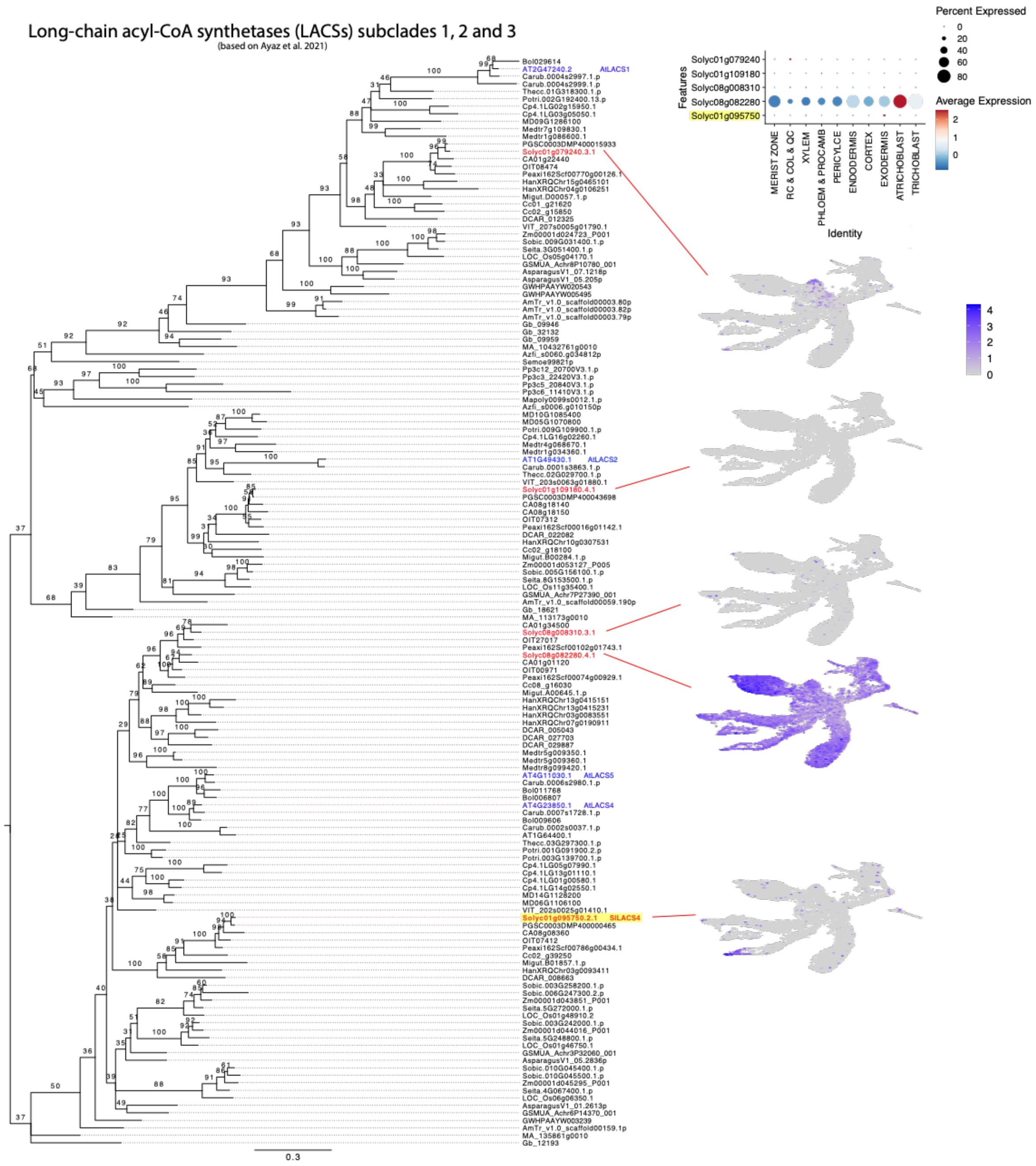

Supp. Fig. 2 (Cont.)

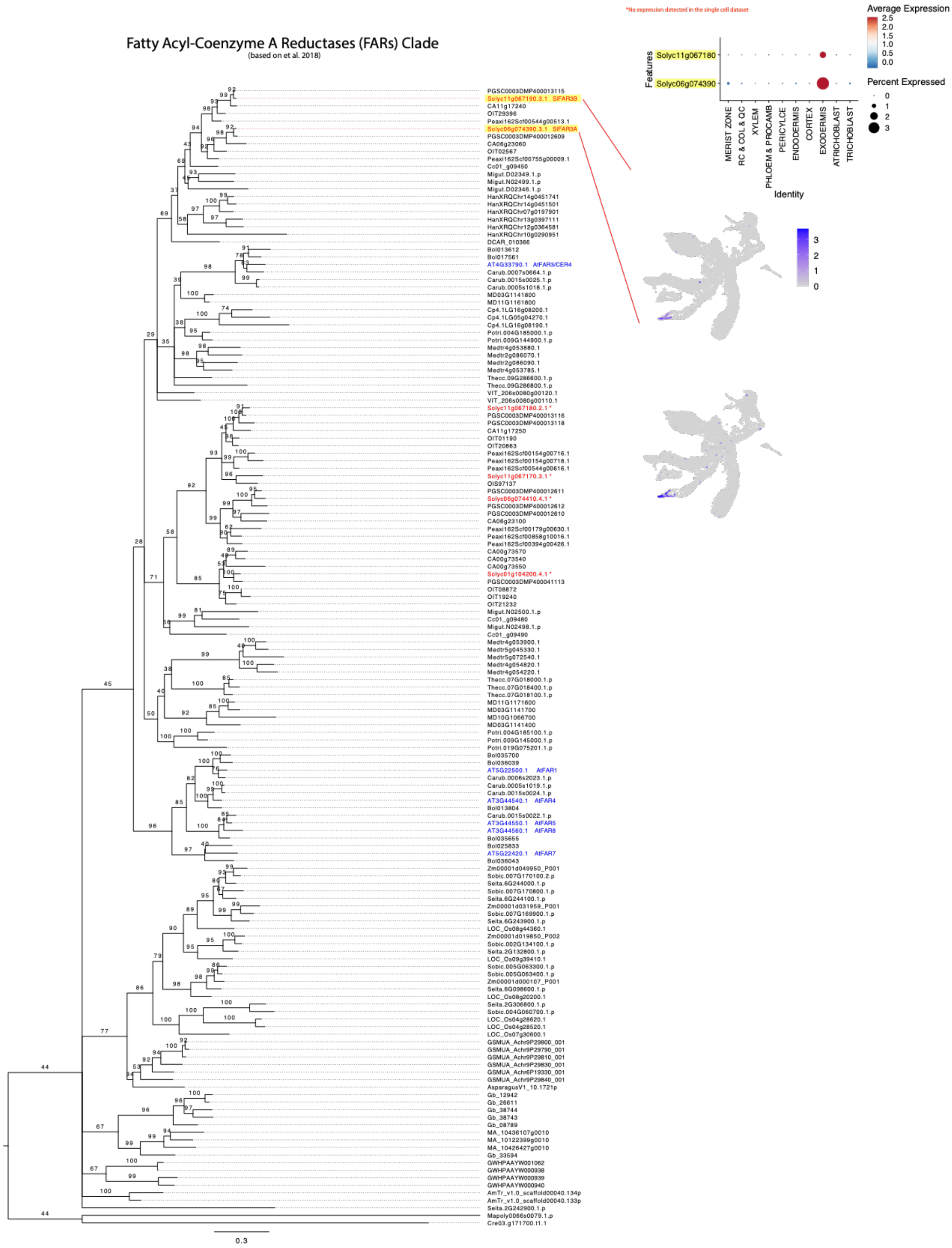

Supp. Fig. 2 (Cont.)

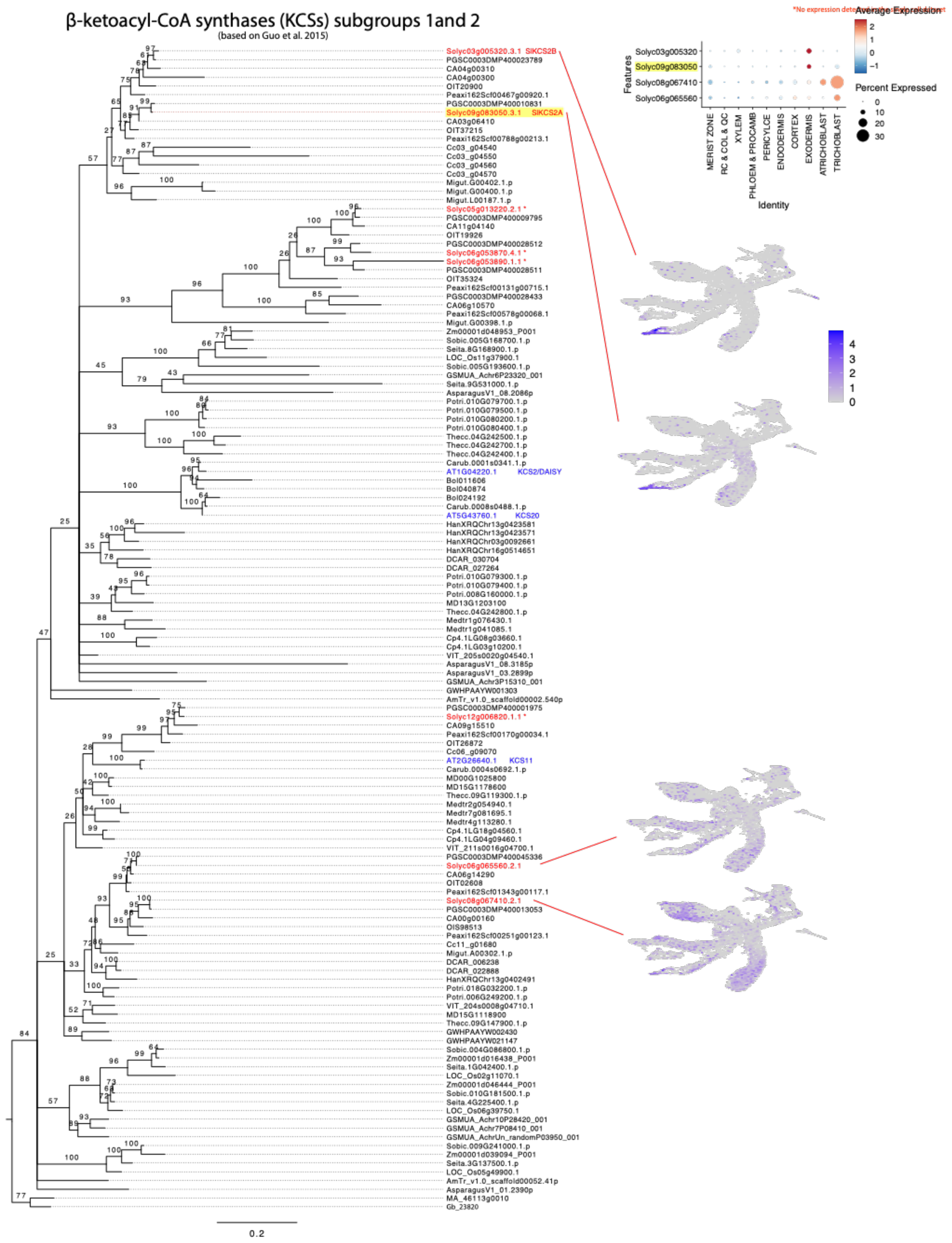

Supp. Fig. 2 (Cont.)

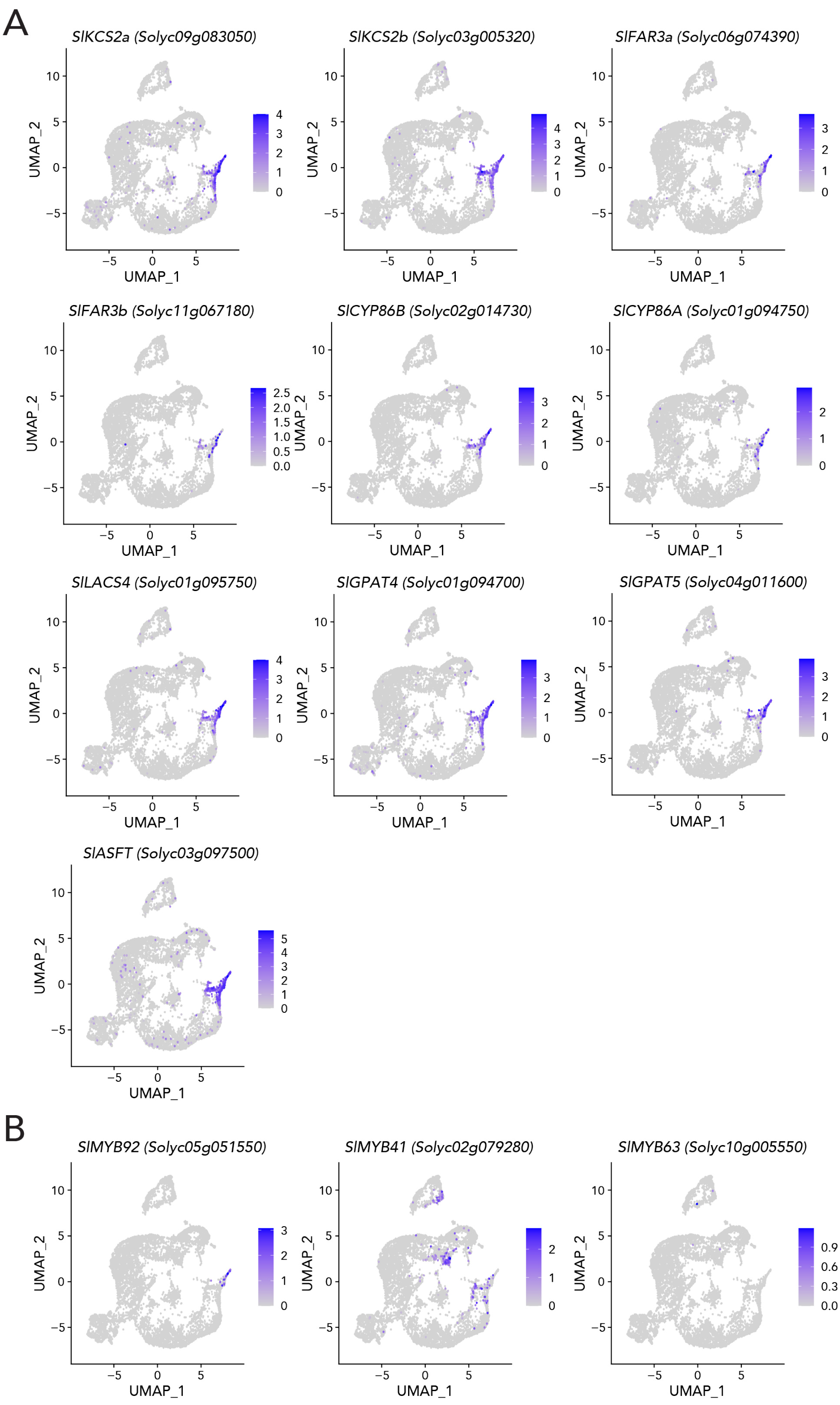

Supp. Fig. 3: Expression of suberin biosynthetic genes and *SlMYB92* is restricted to the mature exodermis. Expression of candidate (A) biosynthetic genes and (B) transcriptional regulators across the UMAP of Endodermis/Exodermis/Cortex single cell populations. The color scales represent log_2-normalized corrected UMI counts.

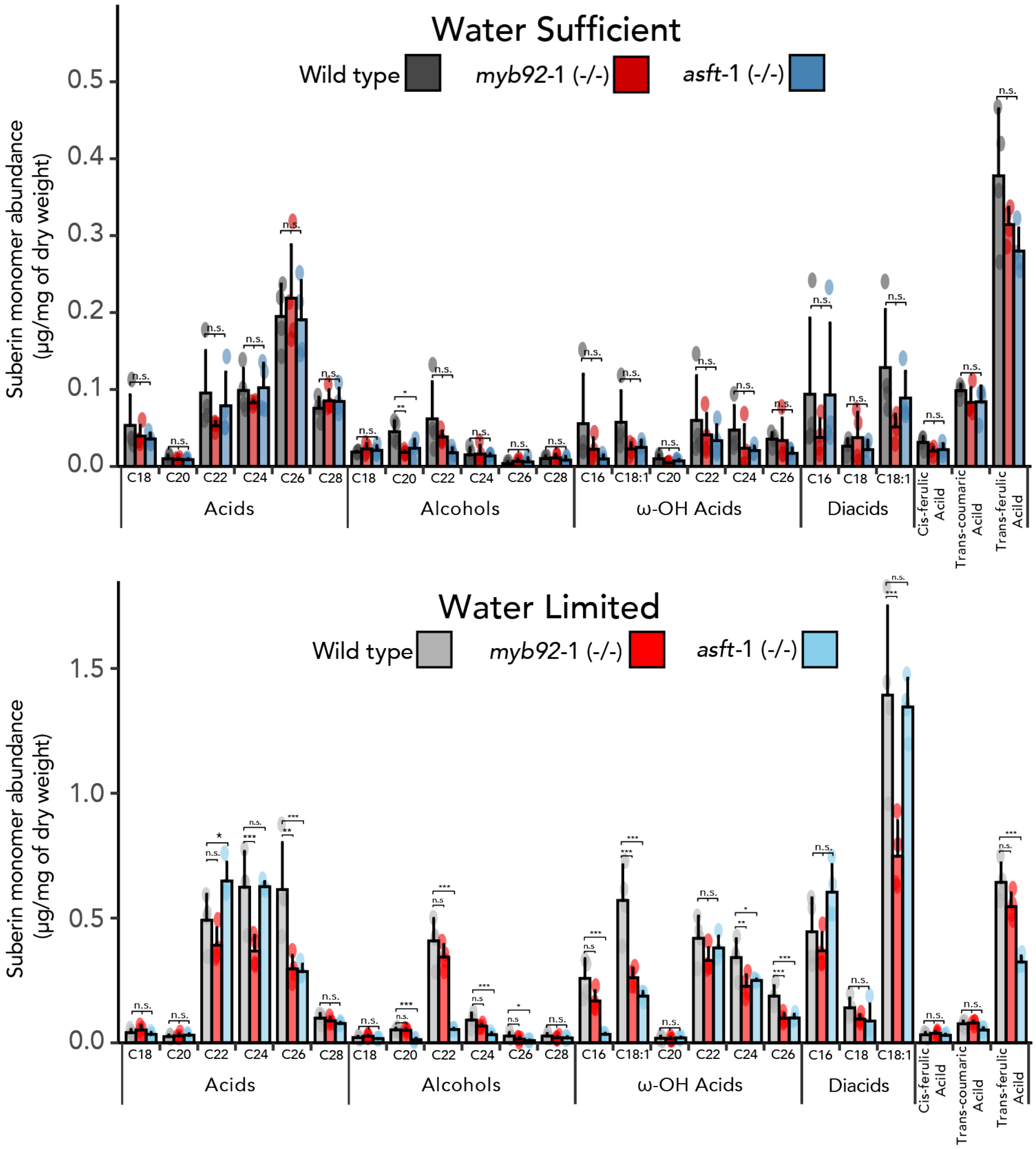

Supp Fig. 4: Suberin monomer breakdown of wild type, *myb92-1,* and *asft-1*. Breakdown of specific monomer abundance of samples under water sufficient (top) and water limited conditions (bottom). Acid: fatty acids; Alcohols: primary alcohols; ω-OH: ω-hydroxy fatty acids; DCA: dicarboxylic fatty acid; Aromatics: ferulate and coumarate derivatives. n = 4, error bars: SD. Asterisks indicate significance vs wild type with One-way ANOVA followed by a Tukey-Kramer post hoc test. Significance codes: '***' <0.001 '**' <0.01, n.s.: "not significant".

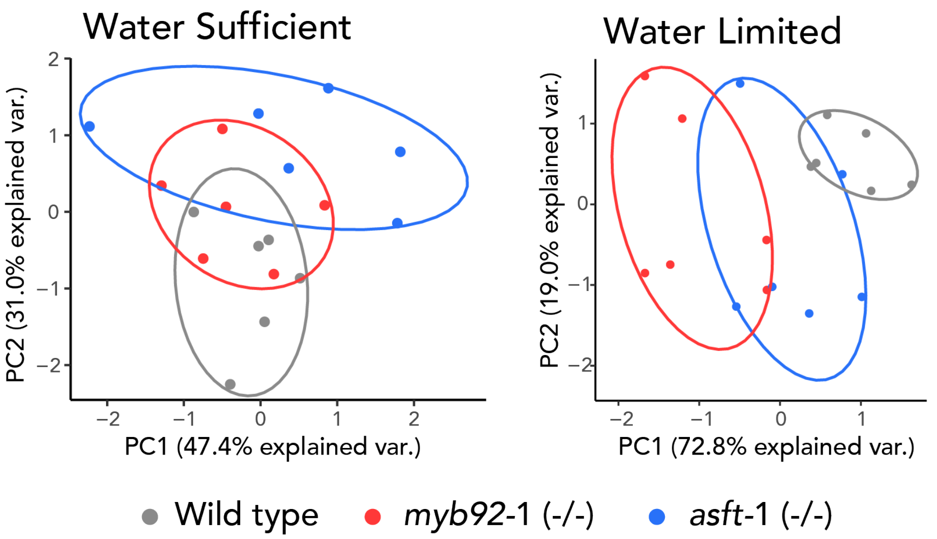

Supp. Fig. 5: Different overall responses to water limitation in *slasft-1* and *slmyb92-1* compared to wild type. Principal Component (PC) analysis of physiological traits of plants grown in water sufficient, and water limited conditions. Each sample is indicated by a dot and colored by the genotype (n=6).
