# Supplementary material for: A suberized exodermis is required for tomato drought tolerance
Source: Nat Plants. 2024 Jan 2;10(1):118–30. doi: 10.1038/s41477-023-01567-x (PMC10808073; doi:10.1038/s41477-023-01567-x)
Supplement: Supplementary file 2 — Reporting Summary [file 41477_2023_1567_MOESM2_ESM.pdf]

nature portfolio

Corresponding author(s): Siobhan Brady
Last updated by author(s): Oct 16, 2023

Reporting Summary

Nature Portfolio wishes to improve the reproducibility of the work that we publish. This form provides structure for consistency and transparency in reporting. For further information on Nature Portfolio policies, see our Editorial Policies and the Editorial Policy Checklist.

Statistics

For all statistical analyses, confirm that the following items are present in the figure legend, table legend, main text, or Methods section.

n/a	Confirmed	
☐	☒	The exact sample size (*n*) for each experimental group/condition, given as a discrete number and unit of measurement
☐	☒	A statement on whether measurements were taken from distinct samples or whether the same sample was measured repeatedly
☐	☒	The statistical test(s) used AND whether they are one- or two-sided<br>*Only common tests should be described solely by name; describe more complex techniques in the Methods section.*
☐	☒	A description of all covariates tested
☐	☒	A description of any assumptions or corrections, such as tests of normality and adjustment for multiple comparisons
☐	☒	A full description of the statistical parameters including central tendency (e.g. means) or other basic estimates (e.g. regression coefficient) AND variation (e.g. standard deviation) or associated estimates of uncertainty (e.g. confidence intervals)
☐	☒	For null hypothesis testing, the test statistic (e.g. *F*, *t*, *r*) with confidence intervals, effect sizes, degrees of freedom and *P* value noted<br>*Give P values as exact values whenever suitable.*
☒	☐	For Bayesian analysis, information on the choice of priors and Markov chain Monte Carlo settings
☒	☐	For hierarchical and complex designs, identification of the appropriate level for tests and full reporting of outcomes
☒	☐	Estimates of effect sizes (e.g. Cohen's *d*, Pearson's *r*), indicating how they were calculated

Our web collection on statistics for biologists contains articles on many of the points above.

Software and code

Policy information about availability of computer code

Data collection	No software was used for data collection.
Data analysis	RNASEQ Trimming: TrimGalore!(v0.6.6); Pseado-alignmnent: Kallisto v0.43.1 and v0.46.2; Clusterin and co-expression: WGCNA R package v1.70; Data handling: R v4.1.3 ;Plotting: ggplot2 v3.3.6. SINGLE CELL: Mapping: cellranger v1.1.0; Processing and analysis: Seurat v4.0.5; TEM images: EM-MENU v4.0 & IMOD v4.11;

For manuscripts utilizing custom algorithms or software that are central to the research but not yet described in published literature, software must be made available to editors and reviewers. We strongly encourage code deposition in a community repository (e.g. GitHub). See the Nature Portfolio guidelines for submitting code & software for further information.

Data

Policy information about availability of data

All manuscripts must include a data availability statement. This statement should provide the following information, where applicable:
- Accession codes, unique identifiers, or web links for publicly available datasets
- A description of any restrictions on data availability
- For clinical datasets or third party data, please ensure that the statement adheres to our policy

Single-cell and bulk RNA-seq data have been deposited at GEO: GSE212405.

Human research participants

Policy information about studies involving human research participants and Sex and Gender in Research.

Reporting on sex and gender	NA
Population characteristics	NA
Recruitment	NA
Ethics oversight	NA

Note that full information on the approval of the study protocol must also be provided in the manuscript.

Field-specific reporting

Please select the one below that is the best fit for your research. If you are not sure, read the appropriate sections before making your selection.

☒ Life sciences ☐ Behavioural & social sciences ☐ Ecological, evolutionary & environmental sciences

For a reference copy of the document with all sections, see nature.com/documents/nr-reporting-summary-flat.pdf

Life sciences study design

All studies must disclose on these points even when the disclosure is negative.

Sample size	We used three biological replicates for RNA-Seq and over 20,000 cells across two rounds on 10X single cell transcriptomics, following common practice in the field. Sample sizes for experiments are indicated in the legends and text. Each analysis was analyzed at least with three biological replicates,and two rounds of experimentation. Biological replicates yielded similar results. No statistical method was used to predetermine sample size. Generally sample size was chosen empirically, based on prior experience of how big a sample size must be to most probably obtain a reproducible, statistically significant result.
Data exclusions	No data was excluded from the analysis.
Replication	At least 3 biological replicates and two independent rounds of experimentation were performed for each experiment to assess quality, repeatability of a procedure, and statistical significance. Number of repeats is provided in the figure legends where appreciate
Randomization	Plants were randomly assigned to water-sufficient or water-limited regimes for the drought experiments. Pots were distributed randomly around the growth chamber and shuffled every other day during watering for the duration of the experiments. When dealing with CRISPR mutants, both in stable and hairy roots, several independent alleles were taken to minimize bias and risk of off-target effects.
Blinding	Transcriptomics experiments were carried out without prior knowledge of experimental outcome, thus, blinding was not applied. Phenotype and confocal analysis were collected and quantified by several researchers independently with similar outcomes. Drought experiment data collection was performed effectively blind by different researchers sampling randomly without prior knowledge of the sample set-up, readings were recorded in the LICOR, and only after collection data was downloaded, parsed out and analyzed.

Reporting for specific materials, systems and methods

We require information from authors about some types of materials, experimental systems and methods used in many studies. Here, indicate whether each material, system or method listed is relevant to your study. If you are not sure if a list item applies to your research, read the appropriate section before selecting a response.

Materials & experimental systems

n/a	Involved in the study	
☒	☐	Antibodies
☒	☐	Eukaryotic cell lines
☒	☐	Palaeontology and archaeology
☒	☐	Animals and other organisms
☒	☐	Clinical data
☒	☐	Dual use research of concern

Methods

n/a	Involved in the study	
☒	☐	ChIP-seq
☒	☐	Flow cytometry
☒	☐	MRI-based neuroimaging
